# Global cascade of kinetic energy in the ocean and the atmospheric imprint

**DOI:** 10.1126/sciadv.adi7420

**Published:** 2023-12-20

**Authors:** Benjamin A. Storer, Michele Buzzicotti, Hemant Khatri, Stephen M. Griffies, Hussein Aluie

**Affiliations:** ^1^ Department of Mechanical Engineering, University of Rochester, Rochester, NY, USA.; ^2^ Department of Physics, University of Rome Tor Vergata and INFN, Rome, Italy.; ^3^ Department of Earth, Ocean and Ecological Sciences, University of Liverpool, Liverpool, UK.; ^4^ NOAA Geophysical Fluid Dynamics Laboratory, Princeton, NJ, USA.; ^5^ Atmospheric and Oceanic Sciences Program, Princeton University, Princeton, NJ, USA.; ^6^ Laboratory for Laser Energetics, University of Rochester, Rochester, NY, USA.

## Abstract

Here, we present an estimate for the ocean's global scale transfer of kinetic energy (KE), across scales from 10 to 40,000 km. Oceanic KE transfer between gyre scales and mesoscales is induced by the atmosphere’s Hadley, Ferrel, and polar cells, and the intertropical convergence zone induces an intense downscale KE transfer. Upscale transfer peaks at 300 gigawatts across mesoscales of 120 km in size, roughly one-third the energy input by winds into the oceanic general circulation. Nearly three quarters of this “cascade” occurs south of 15°S and penetrates almost the entire water column. The mesoscale cascade has a self-similar seasonal cycle with characteristic lag time of ≈27 days per octave of length scales; transfer across 50 km peaks in spring, while transfer across 500 km peaks in summer. KE of those mesoscales follows the same cycle but peaks ≈40 days after the peak cascade, suggesting that energy transferred across a scale is primarily deposited at a scale four times larger.

## Teaser

Global ocean-energy scale-analysis shows ocean weather systems' self-organization and coupling to climate-scale circulation.

## INTRODUCTION

Oceanic general circulation is a central component of Earth’s climate system, without which much of Earth’s surface would be covered by ice (*1*). This circulation comprises motions spanning a wide range of structures and scales from 𝒪(10^4^) km down to 𝒪(1) mm, including coherent jets such as the Gulf Stream and Kuroshio, gyres, and the meridional overturning circulation on basin scales several thousands of kilometers in extent (*2*). Ocean circulation also includes turbulent mesoscale eddies of 𝒪(100) km in size, which pervade the global ocean and contain most of the ocean’s kinetic energy (KE) (*3*).

Mesoscale eddies are the ocean’s equivalent of weather systems, with characteristic timescales of a few months (*4*). Because of their energy and chaotic nature, recent studies (*5*–*11*) have suggested that these eddies may play a substantial role in climate variability that is intrinsic to the ocean, and is distinct from variability due to external forcing of the ocean. Such oceanic internal variability is hypothesized to arise because energy can be transferred between seemingly incoherent mesoscale eddies and the larger scale coherent flow (*12*), which evolves on the long timescales of climate and is directly coupled to it. Below, we provide direct evidence of such transfer.

The KE cascade, conceptualized by Richardson, Kolmogorov, and Onsager (*13*–*16*), is a fundamental process in turbulent flows with profound and far-reaching consequences, including in our everyday lives. The cascade allows the transfer of energy between vastly different length scales and is still an active research topic (*17*, *18*). Because oceanic circulation on scales of 𝒪(100) km and larger is predominantly geostrophic, similar to two-dimensional (2D) flows, it is theoretically predicted to transfer KE upscale (*19*–*21*). However, these theories have been formulated for idealized homogeneous turbulence without boundaries. Flow in the ocean is highly inhomogeneous, with prominent roles played by continental boundaries, bottom topography, winds, and a plethora of multiscale processes.

How important is the upscale cascade pathway of KE from the mesoscales of size 𝒪(10^2^) km? How does it compare to other energy sources and sinks in the oceanic circulation? Answering these questions is important to determine the energy cascade’s potential contribution to climate variability (*6*, *9*, *10*). Quantifying the oceanic energy cascade is also pertinent to a long-standing problem in physical oceanography of how mesoscales gain and lose their energy. Our limited understanding of these sources and sinks contributes to large uncertainties in the oceanic KE budget (*22*).

While we have global estimates of other processes, such as wind forcing (*23*, *24*) and dissipation to bottom drag and wave generation (*25*, *26*), we do not yet have global estimates for the KE cascade. This absence is because the KE cascade is inherently a multiscale process, which requires decomposing the ocean flow at different length scales in a realistic ocean setting. Important progress has been made in this regard, since the seminal work of Scott and Wang (*27*). However, these past investigations of the KE cascade have been limited to analysis of small regions due to their reliance on Fourier transforms in a box (*28*–*31*) or a traditional turbulence approach using so-called “structure function” (*32*). Limitations of these approaches have prevented us from both (i) estimating the global KE cascade rate and (ii) probing length scales larger than a few hundred kilometers. Storer *et al.* (*3*) recently showed that regional analysis misses the gyre-scale components of the oceanic circulation altogether, including a previously unrecognized spectral peak due to the Antarctic Circumpolar Current (ACC). Compared to energy spectra, analysis of the energy cascade within boxes suffers from compounded uncertainty due to the elimination of gyre scales, which introduces uncontrolled errors to the calculation of the cascade even at length scales smaller than the box size (*33*).

Here, we use a recent coarse-graining methodology (*3*, *33*) that frees us from these limitations while conserving energy throughout the global ocean, which is not possible via the approaches of Fourier boxes or structure functions (*3*). At gyre scales, the KE scale transfer shows signatures of the global atmospheric circulation patterns, i.e., Hadley, Ferrel, and polar cells, through five latitudinal bands of alternating upscale and downscale energy transfer due to an exchange with the mesoscales. We find that the atmosphere’s intertropical convergence zone (ITCZ) produces a band of intense downscale KE transfer in the ocean near the equator. The method allows us to calculate global KE spectra at different depths, which show that mesoscales 𝒪(10^2^) km penetrate the entire water column whereas the circulation at gyre scales >10^3^ km weakens notably with depth. We also report the first estimate of 300 GW for the global upscale cascade of mesoscale KE, which demonstrates that it is a substantial energy pathway in the ocean. We find that the seasonal cycle of the mesoscale KE cascade exhibits a characteristic lag time of ≈27 days per octave of length scales such that, in both hemispheres, the KE transfer across 50 km peaks in spring, while transfer across 500 km peaks ≈3 months later, in the summer. KE content at these length scales follows a similar seasonal cycle but with the peak KE occurring ≈41 days after the peak KE transfer, suggesting that the seasonal variability in the KE spectrum is caused, at least in part, by the upscale KE cascade. In this work, we restrict use of the term “cascade” to where the KE transfer is scale local, reverting to the more general “scale transfer” when scale locality has not been determined. In particular, we use cascade when discussing the KE mesoscale transfer and present evidence for its scale locality.

## RESULTS

Our methodology is applied to a 112° state-of-the-art global ocean reanalysis dataset (hereafter “NEMO”) that assimilates data from sea surface temperature, sea level anomaly, in situ temperature and salinity profiles, and sea ice concentration. In contrast to the recent analysis in (*3*), here we use the full velocity, including the geostrophic and ageostrophic components. We also analyze global data from Archiving, Validation, and Interpretation of Satellite Oceanography (AVISO) satellite observations, which are limited to the geostrophic component and are included in the Supplementary Materials (SM). Coarse-graining is performed on a range of length scales, 𝓁, spanning 10 km to the equatorial circumference of Earth (denoted with 𝓁_⊖_ ∼ 40 × 10^3^ km). Details on coarse graining, the dataset, and the presented diagnostics can be found in Materials and Methods. Our results revolve around two key quantities. First is the filtering spectrum (*34*), $\bar{E}(k_{\ell })={\mathrm{dKE}}^{>\ell }/dk_{\ell }$, which measures spectral KE density as a function of length scale, where *k*_𝓁_ = 𝓁^−1^ is the filtering wave number and KE^>𝓁^ is the KE contained at all scales larger than 𝓁. The second diagnostic is KE scale transfer (or cascade), Π_𝓁_, which measures the amount of KE transferred from scales larger than 𝓁 to scales smaller than 𝓁, and is signed so that a positive/negative value indicates a downscale/upscale energy transfer.

### Surface KE spectra

**Fig. 1.**
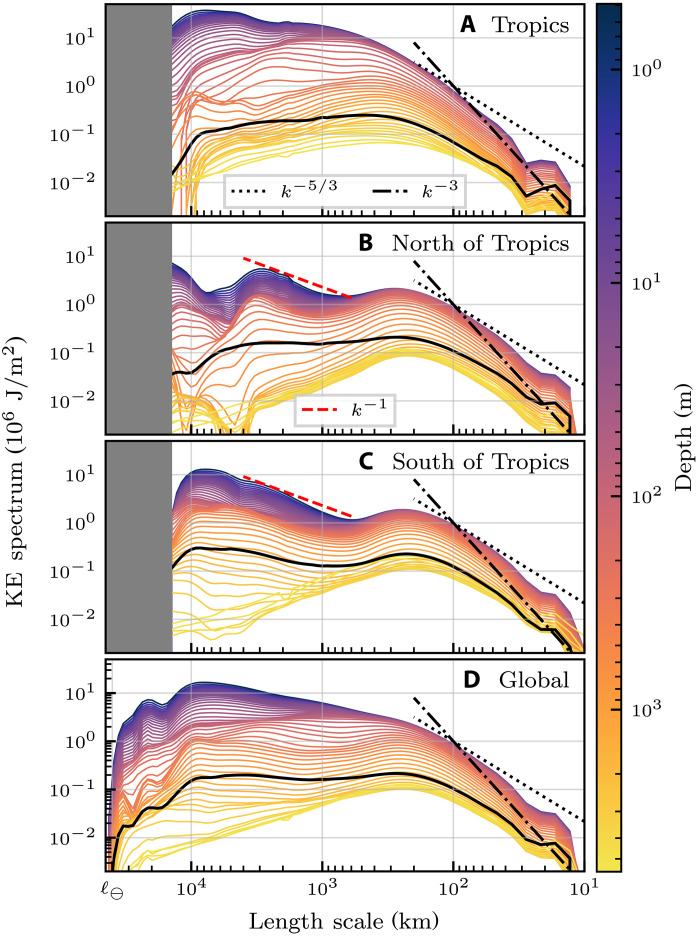
KE filtering spectra. Area-averaged KE spectra (megajoules per square meter; divide by 1025 kg/m^3^ to obtain traditional m^3^/s^2^) as a function of length scale (horizontal axis) and depth (color scale) for four regions of interest (see panel labels). Dashed/dotted black and red lines show various power law slopes, as indicated by the legends. Note that the horizontal axis, 𝓁, decreases to the right. 𝓁_⊖_ denotes the equatorial circumference of Earth, ≈40 × 10^3^ km. All 50 vertical levels of the reanalysis dataset are plotted. For comparison, solid black curves show KE spectra of the depth-averaged flow. See also fig. S1 in the SM, which presents spectra at only a few selected depths. In (**A**) to (**C**), the largest scales (shaded) cannot be restricted to separate regions. (**D**) presents spectra averaged over the entire globe, and so includes the spectra at the largest scales.

Figure 1 presents the area-averaged KE filtering spectra as a function of depth and lateral length scale for the global ocean (“Global”), north of 15°N [“North of Tropics” (NH)], between 15°S and 15°N (“Tropics”), and south of 15°S [“South of Tropics” (SH)]. The surface KE spectra (dark purple lines) in the extratropical hemispheres (NH and SH) broadly follow the same pattern that was found from studying geostrophic velocities (*3*). Namely, there is a mesoscale peak at ≈250 km, a NH gyre peak at ≈3 × 10^3^ km, and an ACC peak at ≈9 × 10^3^ km. Our results then extend those previous findings by considering full model velocity, instead of just the geostrophic velocity, thus allowing us to study the entire global ocean including the tropics.

Consistent with previous studies that focused on regional spectral analyses (*31*, *35*–*39*), the mesoscale spectral scaling lies between *k*^−5/3^ and *k*^−3^ in the global KE spectrum. Because of ageostrophic Ekman flow over the range 10^3^ to 10^4^ km (*12*), the extratropical gyre scales in Fig. 1 (B and C) follow a scaling that is slightly steeper than the *k*^−1^ previously found for the geostrophic velocity (*3*). While the extratropics exhibit an energy minimum separating the mesoscale and gyre-scale peaks, the tropics, which were not analyzed in (*3*), do not have such a spectral energy minimum. Instead, the tropics yield a global ocean surface KE spectrum that monotonically increases with length scale up to scales of ≈10^4^ km.

### KE spectra at depth

Figure 1 provides the first global power spectrum of the ocean as a function of depth (indicated by the color bar). At almost all length scales, spectral KE density decreases monotonically with depth (*37*, *38*), consistent with the surface intensification of the ocean currents. However, the magnitude of such decrease in energy with depth is strongly dependent on length scale. A notable feature in Fig. 1 is how rapidly gyre-scale energy decays with depth compared to energy at the mesoscales. For instance, in Fig. 1D, energy density at 𝓁 = 200 km does not decrease noticeably in the upper ∼100 m, while energy density at 𝓁 = 10 × 10^3^ km decreases by roughly an order of magnitude over the same depth (see also fig. S1D in the SM). Figure 1 (B and C) (and fig. S1, C to F, in the SM) quantifies the gyre scales’ precipitous KE decay with depth. Their KE decreases by a factor of *O*(10) at 500-m depth and of *O*(100) at 2000-m depth compared to the surface, albeit with notable differences between the NH and SH. This surface-trapped gyre-scale motion is consistent, at least in part, with baroclinic Rossby wave adjustment (*40*) and wind-driven Ekman transport, which is restricted to the Ekman layer in the upper ∼100 m (*12*).

### Mesoscales span the water column

Outside of the tropics (Fig. 1, B and C), the mesoscale spectral peak is present at all depths. While their spectral energy density decreases by roughly a factor of 15 from the surface to the abyssal ocean, the mesoscales remain energetically dominant, especially considering that energy density at scales larger than 10^3^ km decreases by two to three orders of magnitude. Figure 1 (B and C) (and fig. S1, C to F, in the SM) quantifies the extent to which mesoscales are barotropic (*41*), with their KE decreasing by a factor of ≈3 at 500-m depth and of 𝒪(10) at 2000-m depth compared to the surface. The observation that the spectral energy per wave number in the mesoscales is on par with or greater than that of the largest scales underscores the dominance of the mesoscales at all depths. Oceans are forced at the surface by winds and buoyancy fluxes, and to maintain equilibrium, some of this energy is transferred to the deeper ocean where it is dissipated at the bottom via friction (*25*, *26*). The mesoscale dominance of the deep ocean spectra highlights their key role in the ocean’s energy dissipation pathways.

### Global scale transfer of KE

**Fig. 2.**
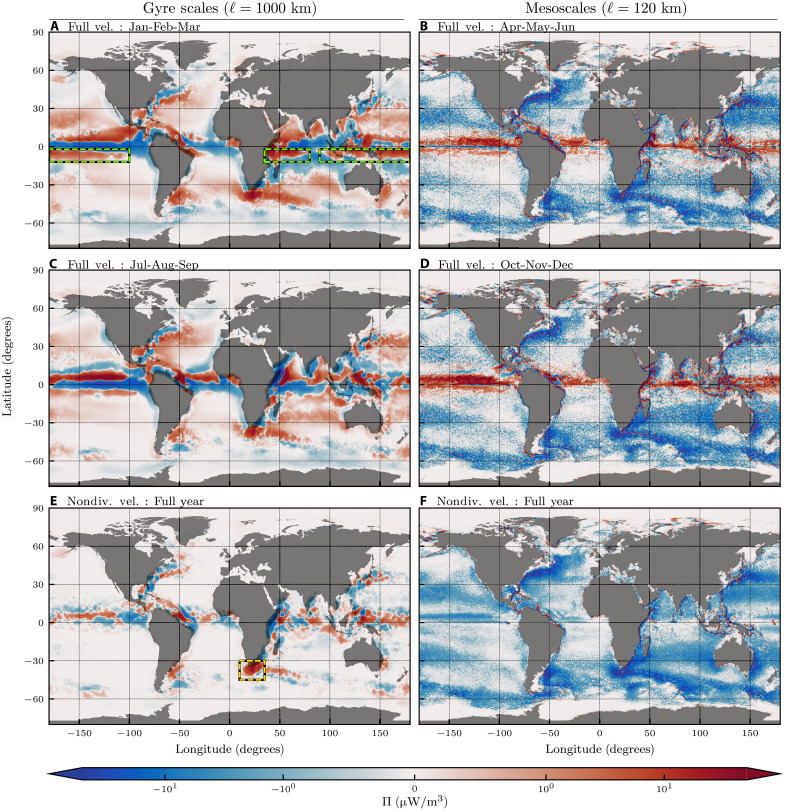
Surface maps of Π_𝓁_. (**A**, **C**, and **E**) 𝓁 = 1000 km and (**B**, **D**, and **F**) 𝓁 = 120 km from 4 years (2015–2018), with a positive (negative) value indicating a downscale (upscale) KE transfer. To highlight ITCZ seasonal imprint on gyre-scale KE transfer and seasonal extrema of mesoscale KE transfer, Π_𝓁_, using full velocity, is averaged over (A) January-February-March (JFM), (B) April-May-June, (C) July-August-September (JAS), and (D) October-November-December. All four seasons are in fig. S5 in the SM. (E and F) Annual averages of Π_𝓁_ but using only the laterally nondivergent velocity. Seasonal maps equivalent to (E) and (F) are in fig. S6 in the SM. All panels share a common color scale. To preserve scale-dependent symmetries, the coarse-grained flow is allowed to be nonzero within a distance 𝓁/2 beyond land boundaries (gray mask), consistent with the uncertainty principle (see Materials and Methods). (A) Green dashed boxes highlight the Southern ITCZ red branch imprint that occurs during JFM. A corresponding ITCZ imprint can be seen north of the equator during JAS (C) in all basins. (E) Orange dashed box highlights strong downscale KE transfer from geostrophic flow shear between the Agulhas and ACC.

Figure 2 provides the first global maps of the surface KE scale transfer, Π_𝓁_, across 𝓁 = 1000 km (gyre scales) and 𝓁 = 120 km (mesoscales), averaged over 2015–2018 (see also fig. S8 in the SM using satellite data). Positive Π_𝓁_ values (red) indicate a downscale transfer of KE from scales larger than 𝓁 to those smaller than 𝓁, while negative Π_𝓁_ values (blue) indicate an upscale transfer across 𝓁. These geographic maps highlight a key advantage of the coarse-graining methodology over traditional approaches using Fourier or structure functions: we can retain spatial information while concurrently diagnosing processes at different scales. The maps in Fig. 2 allow us to associate the scale transfer of KE with flow properties in different regions, as we discuss below. To highlight seasonal trends, gyre-scale KE transfer maps (Fig. 2, A and C) are averaged over winter and summer months, while the mesoscale energy transfer across 120 km are averaged over spring and autumn when scale transfer across that scale is at an extremum (see fig. S5 in the SM for all seasons). Figure 2 (E and F) shows full-year means of the KE scale transfer but only due to the laterally nondivergent component of the ocean currents, which includes geostrophic motions but excludes the divergent Ekman flow (see fig. S6 in the SM for seasonal maps). To preserve physical properties (symmetries) at different scales, the coarse-grained flow is allowed to be nonzero within a distance 𝓁/2 beyond the continental boundary over land (*3*, *33*, *42*). Forfeiting exact spatial localization to gain scale information is theoretically inevitable because of the uncertainty principle (see Materials and Methods).

**Fig. 3.**
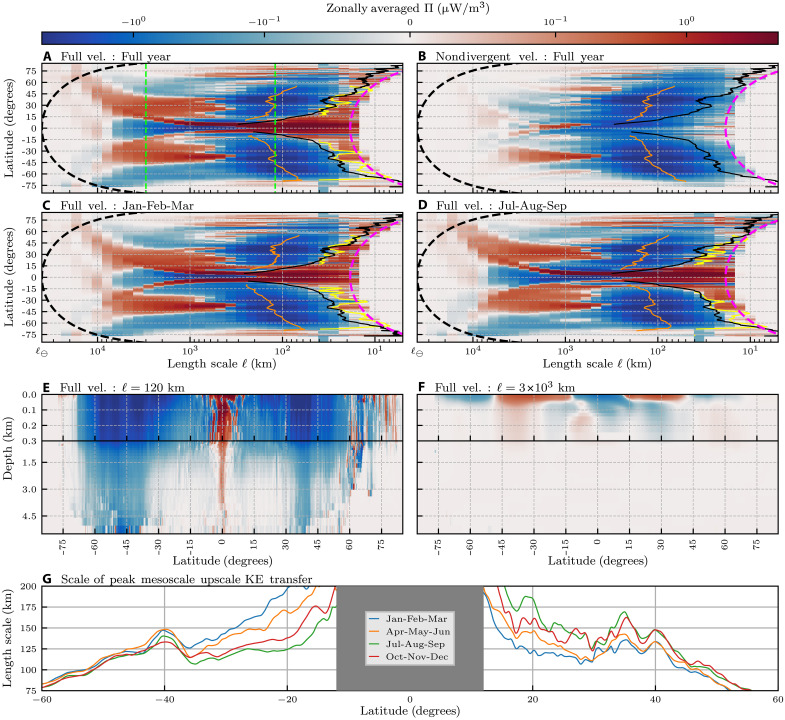
Structure of the KE scale transfer. (**A** to **D**) The zonally averaged surface KE scale transfer as a function of latitude and scale: (A) using full velocities and averaged over all months, (B) using only laterally nondivergent velocities and averaged over all months, (C) using full velocities and averaged over JFM, and (D) using full velocities and averaged over JAS. Negative (positive) values indicate an upscale (downscale) transfer of energy. 𝓁_⊖_ denotes the equatorial circumference of Earth, ∼40 × 10^3^ km. Thick dashed black lines denote the circumference of a line of constant latitude. Thick dashed pink lines denote the zonal width of two grid-points. Straight dashed green lines in (A) denote the length scales analyzed in (**E**) and (**F**). In (A) to (D), orange lines show the scale with greatest magnitude upscale KE transfer [“mesoscale peak,” (G)], yellow lines show the small-scale transition to downscale transfer, and black lines show the length scale at which the Rossby number equals 0.1. (E and F) Latitude and depth for 𝓁 = 120 km and 𝓁 = 3 × 10^3^ km [dashed green lines in (A)]. Note that the vertical axis is split at 300-m depth, so that the upper 300 m are emphasized. The color bar is the same for all panels. (**G**) Mesoscale peak for each of the seasonal bands, smoothed using a 3° moving average. Figure S3 reproduces (C) and (D) with additional annotations.

Figure 3 complements the scale transfer maps in Fig. 2 by showing the zonally (east-west) averaged scale transfer as a function of latitude, length scale, and depth. Figure 3A presents time-mean surface KE scale transfer as a function of latitude and scale. In the SM, fig. S3 is an annotated version of Fig. 3, and fig. S7 presents the same analysis from the AVISO satellite product.

### Mesoscale KE transfer

Maps of KE scale transfer at the mesoscales (Fig. 2, B and D) show the pervasiveness of the upscale KE transfer, which dominates the extratropical global ocean and is consistent with geostrophic turbulence theory (*12*, *21*). The upscale transfer is most intense in dynamically active regions, including the Gulf Stream, Kuroshio, and the ACC, because of strong mesoscale eddy activity and large baroclinic growth rates (*43*). In contrast, equatorial regions, which are not constrained by geostrophy, show a prominent downscale KE transfer (red) across a scale of 120 km in Fig. 2 (B and D). Comparing maps in Fig. 2 (B and D), we find indications that the mesoscale upscale KE transfer across 120 km is stronger during the local spring. This behavior is particularly noticeable in the subtropics, such as in the Kuroshio region and around Australia. Figure 2Fshows that the laterally nondivergent flow component, which excludes upwelling/downwelling motions, exhibits a more pronounced upscale transfer, including in the tropics.

In Fig. 3 (A to D), the upscale transfer (blue) is prominent over a wide range of mesoscales, and spans roughly (65°S,15°S) and (15°N,55°N) (see also fig. S7 in the SM). In each panel, we outline three length scales of interest: (i) the length scale with the strongest mesoscale upscale KE transfer (orange, “mesoscale peak”), (ii) the scale at which the KE transfer transitions to downscale (black, “downscale transition”), and (iii) the length scale corresponding to Rossby number of 0.1 (yellow, 𝓁_Ro=0.1_), with larger values of Rossby number indicating a reduced influence from Earth’s rotation (see Materials and Methods).

The scale of the peak upscale KE transfer (orange lines in Fig. 3, A to D, also shown in Fig. 3G) ranges from ≈200 km near the equator to ≈80 km near the poles. The peak scale generally decreases polewards except in strong current systems (see Fig. 3G and fig. S7C in the SM). There is a notable increase in the peak scale at 40°S associated with the ACC, and two smaller increases at ∼35°N and 40°N, associated with the Gulf Stream and Kuroshio. In these strong currents, the mesoscale nonlinear interactions are more intense (Fig. 2, B and D), transferring energy upscale over a wider range of scales. This latitudinal dependence of the length scale corresponding to the peak upscale KE transfer is consistent with previous studies that used Fourier analysis to compute KE scale transfer (*27*, *31*, *38*, *43*), with our analysis here covering the global ocean without being confined to regional boxes.

Transition from upscale to downscale KE transfer in Fig. 3 tracks 𝓁_Ro=0.1_ relatively well, suggesting that the transition to downscale KE transfer is driven by unbalanced motions, consistent with previous work (*32*, *38*, *44*). Excluding the irrotational flow, scale transfer from the laterally nondivergent flow at these small scales seems to exhibit an upscale transfer in Fig. 3B. Given that the resolution of the dataset used here is 112° (∼9 km at the equator), this transition can only be considered marginally resolved and submesoscale permitting at best (*36*) and should be further investigated with higher resolution data.

The tropics are the only latitudes that demonstrate a persistent downscale transfer (red) over mesoscales (𝓁 < 500 km). As 𝓁 increases from 𝒪(100) km to scales >1000 km over the tropics in Fig. 3, the downscale energy transfer bifurcates into two intense off-equatorial branches (red) that lie within ≈ ±10° of the equator (“red branches” in fig. S3). The two branches have a distinct north-south asymmetry, with the northern band exhibiting a stronger downscale transfer due to the asymmetric seasonal migration of the ITCZ as we elaborate below.

### Gyre-scale KE transfer and the atmospheric imprint

Maps of the KE transfer across gyre scales in Fig. 2 (A and C) and their zonal average in Fig. 3A reveal an imprint of the global atmospheric circulation. This imprint is most clear from Fig. 3A, where we find five “cells” of alternating upscale and downscale transfer at the ocean surface (c.f. “Atmospheric Cell Imprint” in fig. S3). Each cell spans ≈30° in latitude. They are centered at the equator, horse latitudes (30°N and 30°S), and polar fronts (60°N and 60°S), which mark the transition zones between the atmospheric Hadley, Ferrel, and polar cells.

This atmospheric signature on the gyre-scale oceanic KE scale transfer can be explained by the wind-driven Ekman transport within those bands (*12*). Zonal surface wind stress (τ_λ_) induces meridional (north-south) Ekman velocity ($u_{\phi }^{E}$; see Materials and Methods), such that a meridionally alternating wind direction produces meridionally alternating divergent and convergent flows within the ocean’s Ekman layer (top ∼100 m). These flows would respectively “stretch” and “compress” oceanic motions, leading to KE transfer to larger and smaller scales, respectively. We shall see below that much of the gyre-scale transfer occurs because of energy exchange with the mesoscales. A concept worth emphasizing is that energy can undergo an inertial scale transfer of KE in the presence of coherent convergent or divergent flow structures such as in shocks, rarefaction waves, and fronts (*45*–*48*). While KE scale transfer due to convergent and divergent (unbalanced) motions has been analyzed at the submesoscales in regional models (*44*, *49*), the results presented here are the first to show scale transfer due to the convergent and divergent Ekman flow at gyre scales. To reinforce this interpretation, Figs. 2E and 3B present Π_𝓁_ arising solely from the laterally nondivergent (toroidal) flow component, which lacks the necessarily divergent Ekman flow component and the associated atmospheric-cell pattern. Further support for this interpretation comes from examining the depth profile of the gyre-scale KE transfer (Fig. 3F, discussed in the next paragraph), where we find that the alternating upscale and downscale transfer bands are localized to the top ≈100 m in the ocean, which is approximately the same depth as the Ekman layer. From Fig. 3 (A, C, and D), we note a north-south asymmetry in the gyre-scale KE transfer poleward of 45°. South of 45°S, the upscale transfer (blue) persists to larger scales and with higher intensity than its NH counterpart. This behavior is attributed to continental boundaries, which constrain the upscale transfer (blue) north of 45°N unlike in the Southern Ocean.

#### Depth profiles of KE scale transfer

Figure 3 (E and F) presents the latitude-depth profiles of Π_𝓁_ for 𝓁 = 120 km and 𝓁 = 3 × 10^3^ km, which represent the mesoscale and gyre-scale KE scale transfer signals in Figs. 2 and 3A. The mesoscale transfer (Fig. 3E), in addition to having strong intensity, penetrates the entire water column. The scale transfer is surface intensified (note the log scale in the color bar), but there is a clear upscale transfer down to 5-km depth, especially between (60°S,30°S) and (30°N,45°N), which are the approximate latitudes of the ACC, Gulf Stream, and Kuroshio. This result provides evidence that the upscale mesoscale transfer has a substantial barotropic component, in accord with the theory of geostrophic turbulence (*50*). In contrast, the gyre-scale KE transfer (Fig. 3F) is mostly surface trapped to the upper 100 m of the ocean, where wind effects are most pronounced. Figure 3F also demonstrates that the downscale/upscale KE transfer due to Ekman flow convergence/divergence near the surface is not cancelled by the return Ekman flow divergence/ convergence at greater depth. This lack of cancellation is because the KE scale transfer (Eq. 5 in Materials and Methods) arises from flow strain at scales >𝓁 acting against stress from motions at scales <𝓁. These subscale motions are much stronger near the surface than at depth (Fig. 1 and fig. S1), underscoring the importance of vertical inhomogeneity in the oceanic scale transfer of KE.

In the equatorial region, we can see clearly in Fig. 3E that mesoscale KE transfer differs substantially from other latitudes. It is characterized by strong downscale KE transfer, which penetrates several kilometers into the water column. This downscale transfer may be related to shear induced by alternating equatorial deep jets (*51*, *52*), with the meridionally broader but shallower downscale transfer possibly owing to the subsurface countercurrents. Further analysis of this phenomenon is perhaps better investigated using regional modeling of the equatorial region at higher vertical resolutions.

#### Departures from geostrophic theory

The laterally nondivergent flow, which includes geostrophic (balanced) motions, exhibits a general tendency to transfer KE upscale (blue) at all latitudes and depths for scales <1000 km (c.f. Figs. 2F and 3B). This mesoscale upscale KE transfer is consistent with idealized geostrophic turbulence theory (*12*, *50*), which neglects nonideal effects from winds, regional inhomogeneity, and boundaries. As we find here, these nonideal effects, which exist in the realistic NEMO global ocean reanalysis and AVISO satellite data, can become important at gyre scales. Geostrophic flow at the gyre scales (Fig. 2E and fig. S8A) exhibits downscale transfer (red) in regions of strong shear, including the flanks of the Gulf Stream and Kuroshio [see also (*33*)]. This behavior is perhaps clearest in the Agulhas retroflection (orange box in Fig. 2E), where the westward Agulhas current turns back on itself because of strong shear from the eastward flowing ACC. The effect can also be seen from the zonally averaged scale transfer in Fig. 3B, for 𝓁 > 500 km between (45°S,30°S), where the laterally nondivergent velocity field produces a local net downscale KE transfer.

**Fig. 4.**
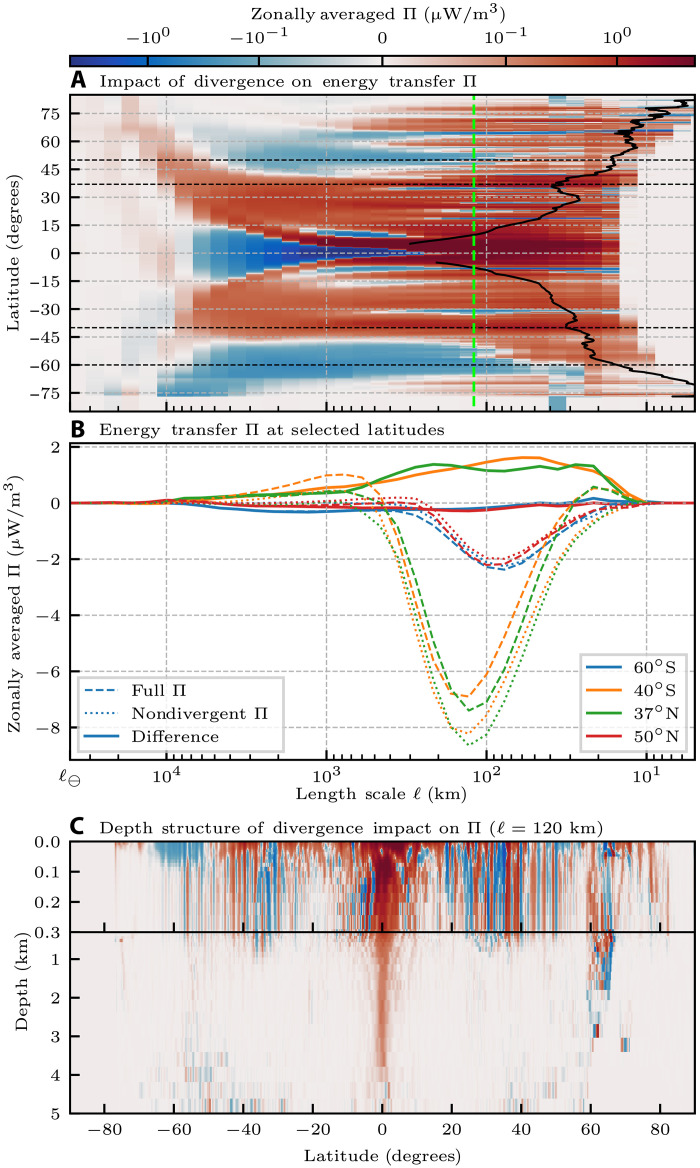
Impact of divergent flow on energy transfer. This extends Fig. 3 by highlighting the impact of laterally divergent motions on KE transfer at all scales. (**A**) subtracts Fig. 3B from Fig. 3A to exclude contributions from self-interactions among nondivergent (geostrophic) flow, thereby showing KE transfer involving interactions with the laterally divergent flow. (**B**) Transfer at select latitudes indicated by horizontal dashed black lines in (A). (**C**) is analogous to 3E. The black contour line in (A) is the same as in Fig. 3A and shows the length scale at which the Rossby number is 0.1. The green dashed line in (A) shows the length scale used in (C).

Direct evidence for the existence of energy exchange between gyre scales and mesoscales can be gleaned from Fig. 4. It shows the KE scale transfer involving interactions with the laterally divergent flow, which includes the gyre-scale Ekman transport. Such KE scale transfer excludes interactions of the geostrophic (laterally nondivergent) flow with itself shown in Fig. 3B, which is generally characterized by a dominant upscale KE transfer. We emphasize that this Ekman-induced scale transfer is measured directly from the ocean currents, and not indirectly inferred from the wind stress curl. Figure 4A can be obtained simply as the difference of Fig. 3A and Fig. 3B, which is possible because the energy transfer term Π (Eq. 5 in Materials and Methods) can be decomposed exactly into contributions from the laterally divergent/nondivergent flow and their nonlinear interactions (see Materials and Methods).

After removing the geostrophic upscale cascade (Fig. 3B), Fig. 4 reveals that the alternating bands of Ekman-induced energy transfer extend from the gyre scale to the mesoscales. The persistent scale transfer of energy spanning the range of scales 𝒪(10^4^) to 𝒪(10^2^) km and smaller in Fig. 4 (A and B) demonstrates the ability of gyre-scale motions to exchange KE with the mesoscales. At gyre scales (>1000 km), the flow experiences an effective stress from subgyre-scale motions (Eq. 5 in Materials and Methods), which is mainly due to the mesoscales (<500 km) where most of the energy resides. A convergent gyre-scale flow in the subtropics amplifies the KE of mesoscale eddies, much like a piston pushing (adiabatically) on a gas amplifies the KE of gas molecules (i.e., gas temperature or internal energy). Energy transfer occurs because a converging piston does work against the stress (pressure) exerted by molecules via pressure dilatation [−*P*  div (**u**)], which appears in the internal energy budget of a compressible flow (*47*). Similarly, a divergent gyre-scale flow at latitudes (45°,70°) attenuates the KE of mesoscale eddies, much like a piston rarefying a gas attenuates the KE of gas molecules. Such an analogy between mesoscale eddies and gas molecules can be justified thanks to the seeming separation of scales between the gyre-scale spectral peak and the mesoscale peak shown in (*3*) and also in Fig. 1 (B and C). In analogy with the gas molecules’ KE increase, gyre-scale compression brings mesoscale eddies closer together and leads to a gain in mesoscale KE due to enhanced self-advection (*15*). Given the wide scale separation, gyre-scale convergence is probably inefficient at amplifying the mesoscales by vortex stretching. We present this piston-pressure framework not as an established fact but as a proposed theory for how the gyre scales and mesoscales can interact in a manner that explains the energy transfer measured in Fig. 4.

Figure 4B shows that the gyre-scale–mesoscale transfer due to gyre-scale convergence/divergence at select latitudes is a substantial fraction of the upscale mesoscale cascade. When these different processes are combined into the full scale transfer in Fig. 3A, the upscale mesoscale cascade masks the gyre-scale–mesoscale transfer.

Figure 4C examines the depth profile of KE transfer shown in Fig. 4A at 120 km. In the extratropics, we see that the alternating latitudinal bands of KE transfer, while being primarily localized to the upper ≈100 m, exhibit columnar features that penetrate to ≈300-m depth. This is similar to the depth over which the upscale mesoscale cascade is strongest in Fig. 3E.

### Seasonality

We now report on seasonal variations in the KE scale transfer at both gyre scales and mesoscales.

#### Gyre scales - atmospheric cells

There are three prominent seasonal trends in the gyre-scale (1 × 10^3^ to 10 × 10^3^ km) transfer of KE in Figs. 2 (A and C) and 3 (C and D). First, consider the five latitudinal bands associated with the atmospheric cells (c.f. “Atmospheric Cell Imprint” in fig. S3), which are known to strengthen and shift equatorward during the winter of each hemisphere (*53*). This seasonality is clearest in the zonally averaged scale transfer in Fig. 3 (C and D), which shows that the two extratropical bands at scales >1000 km (one red and one blue) shift equatorward by a few degrees during the winter of each hemisphere. Note also (Fig. 2, A and C) the seasonality of KE transfer associated with the Indian monsoon winds, which have a southward/northward component during winter/summer (*53*). The resultant Ekman ocean flow (Eq. 9 in Materials and Methods) is westward/eastward, which yields a downscale KE transfer (red) off the eastern coasts of the Arabian peninsula, Horn of Africa, and Indian subcontinent in winter due to convergence toward land and an upscale KE transfer (blue) during summer due to divergence away from land.

#### Gyre scales - red branches and the ITCZ imprint

The second prominent seasonal trend is the feature that we term red branches (c.f. fig. S3). Figure 3 (C and D) presents two large-scale off-equatorial downscale signals: one equatorward of 10°N, which is present for much of the year but is strongest during July-August-September (JAS), another equatorward of 10°S that is present during January-February-March (JFM). The red branches are caused by an interplay between the ITCZ and Ekman transport as we shall now explain.

The ITCZ is a latitudinal band at which the northeast and southeast trade winds from the two Hadley cells converge near the equator and yield intense tropical rainfall within 10°S to 10°N (*54*, *55*). In the zonal average, the ITCZ is located in the NH for most of the year but is strongest during the boreal summer, typically shifting to the SH only during the austral summer (*56*, *57*). The red branches in Fig. 3 (C and D) at scales 𝒪(10^3^) km align with the ITCZ latitudes (*54*). These red branch latitudes can also be seen from the scale transfer maps in Fig. 2 (A and C), which show intense downscale KE transfer (red) just off of the equator. During the SH summer, we see a red latitude band (green box in Fig. 2A) just south of the equatorial Pacific and Indian oceans. During the NH summer (Fig. 2C), an intense downscale transfer band can be seen just north of the equator in the Pacific, Atlantic, and Indian oceans.

**Fig. 5.**
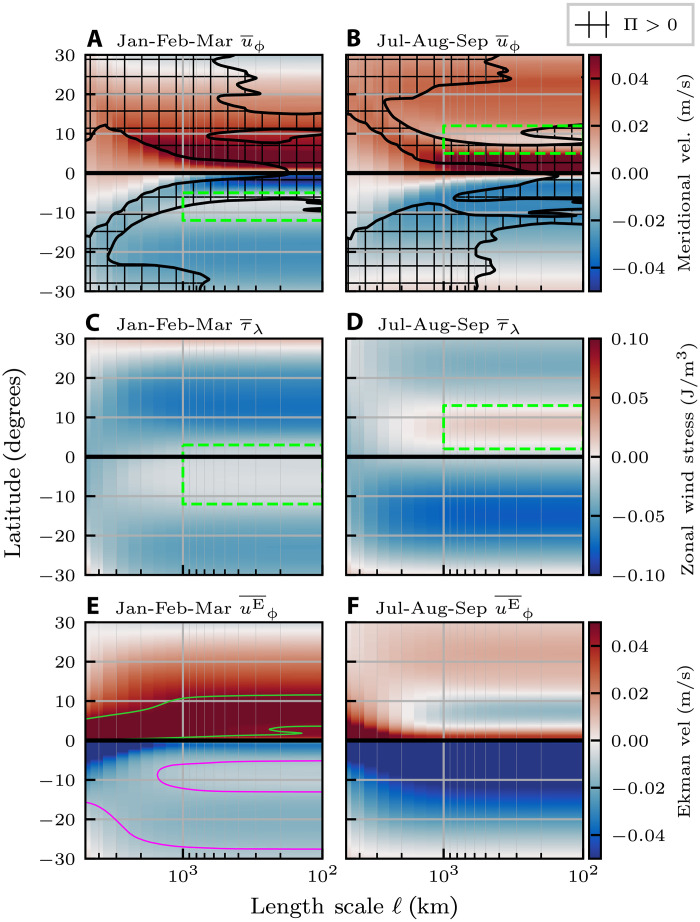
ITCZ scale-transfer mechanism. Zonal means of (**A** and **B**) meridional surface velocity (*u*_ϕ_) from NEMO, (**C** and **D**) zonal wind stress (τ_λ_), and (**E** and **F**) associated meridional Ekman velocity $u_{\phi }^{E}$ (Eq. 10) within 30° of the equator for filter scales larger than 100 km. In (A) and (B), cross-hatching indicates latitudes at which the zonal-mean surface KE scale transfer Π_𝓁_ is positive (i.e., downscale). Green boxes are included to highlight regions of weak meridional transport and zonal wind stress. (E) Green lines show the 4.7 cm/s contour, and magenta lines show the −1.2 cm/s contour. Wind data are from ERA5 reanalysis wind components used in the NEMO ocean reanalysis.

The ITCZ, being a band of weak zonal winds, is associated with a sudden slowdown in the poleward Ekman flow leaving the equator (green boxes in Fig. 5, A and B). Such a slowdown is akin to a hydraulic jump in a river encountering an obstacle, causing surface flow convergence and downwelling. Figure 5 (A and B) shows the zonally averaged meridional velocity from NEMO as a function of latitude and scale during JFM (Fig. 5A) and JAS (Fig. 5B). Figure 5 (A and B) overlay the same downscale branches (cross-hatching) seen in Fig. 3 (C and D) (red branches). In the SH, the poleward flow weakens between 5°S and 10°S during JFM (green box in Fig. 5A), with a similar weakening present in the NH during JAS (green box in Fig. 5B). Slowdown in the poleward flow is caused by the zonal relative wind stress in Fig. 5 (C and D), which show that the typically westward wind stress weakens in the ITCZ band. Recall that meridional Ekman velocity, $u_{\phi }^{E}\propto -\tau_{\lambda }/f$ (Eq. 10 in Materials and Methods), is proportional to the zonal wind stress, τ_λ_, so that the change in sign of the Coriolis parameter, *f*, across the equator induces divergence under a westward wind, and the small magnitude of *f* near the equator means that even modest wind stress can produce a substantial flow. The Ekman flow associated with the wind stress is shown in Fig. 5 (E and F).

We can also explain why the NH red branch is still present during JFM (Fig. 3C), when the ITCZ is mostly south of the equator. The northern branch is perennial because the ITCZ (zonally averaged) does not migrate as far south in the boreal winter as it migrates north in the boreal summer, maintaining an oceanic imprint north of the equator. Figure 5E shows contours of weak Ekman flow: 10 cm/s (green) and −2.5 cm/s (magenta). During JFM, the NH poleward Ekman flow leaving the equator (Fig. 5E) undergoes an acceleration followed by sudden slowdown (green contour lines at the equator and at ≈5°N), resulting in a convergent flow and a downscale KE transfer. Note that exactly at the equator, we always have an upscale KE transfer at gyre scales (blue in Fig. 3). This transfer is due to Ekman flow divergence from a sharp southward to northward flow reversal at the equator caused by a reversal in the direction of the Coriolis force (Eq. 7 in Materials and Methods).

#### Gyre scales - “blue tongue”

The third gyre-scale seasonal trend is also caused by the ITCZ, albeit indirectly, appearing during a hemisphere’s summer (Fig. 3, C and D) as an upscale “tongue” (blue). It emerges at scales larger than 500 km in the subtropics, poleward of the ITCZ’s red branch ocean imprint, between latitudes 5° and 20° (c.f. “blue tongue” in fig. S3). From the map in Fig. 2A, we see that during JFM, the blue tongue is mostly due to an upscale transfer in the Indian Ocean, just south of the downscale (red) band, extending from Madagascar to Australia. Figure 2C shows that during JAS, the blue tongue in the NH is due to an upscale KE transfer that is prominent in the northern tropics of the Atlantic and Pacific.

Blue tongue regions are caused by the Ekman flow’s poleward reacceleration after encountering the slowdown caused by the ITCZ. In Fig. 5, we can see that poleward of the slowdown (green boxes in Fig. 5, A and B), the flow speed increases. This behavior can also be understood from magneta contours of the Ekman flow in Fig. 5E, where the southward flow at ≈10°S increases in speed before slowing down again at ≈30°S. Such reacceleration between 10°S and 20°S is associated with a divergence and Ekman upwelling, manifested as a summer blue tongue in Fig. 3 (C and D).

#### Mesoscale cascade peak

The mesoscale cascade is qualitatively consistent across the four seasons. Figure 3G shows the length scale with the strongest mesoscale inverse cascade as a function of latitude for each season. While the seasonal trends are clearer in the southern hemisphere, we can see that in both hemispheres, the mesoscale cascade peak scale is largest during the local summer and exhibits the greatest seasonal variation in the subtropics, between 15° and 30°.

#### Seasonality of mesoscale KE spectrum and the cascade

**Fig. 6.**
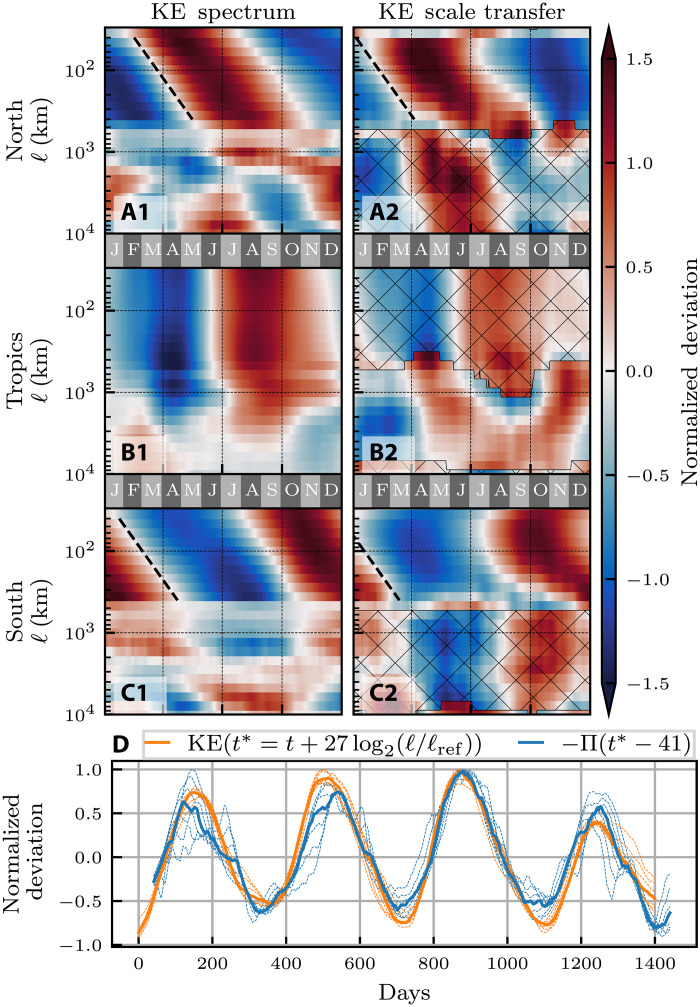
Spectral advection of surface KE and Π_𝓁_. For each scale, panels show normalized variation (*z*-score) of the time series of (**A1**, **B1**, and **C1**) the KE filtering spectrum, $\bar{E}(k_{\ell })$ as in (*3*) and (**A2**, **B2**, and **C2**) the KE scale transfer Π_𝓁_. A 60-day moving average is applied to remove high-frequency signals (*3*). For KE, red indicates where KE is higher than the time mean. For Π_𝓁_, red indicates where the magnitude of the scale transfer is higher than the time mean, with hatching indicating where the scale transfer is downscale. The full time series has been averaged onto a single “typical” year profile. Dashed black lines indicate a spectral advection speed of 27 days per octave. In (A2), (B2), and (C2) showing Π_𝓁_, the dashed black lines are plotted 41 days earlier in the year than the corresponding lines in (A1), (B1), and (C1) showing $\bar{E}(k_{\ell })$. (**D**) $\bar{E}(k_{\ell })$ (orange) and −Π_𝓁_ (blue) for length scales between 60 and 400 km in the NH. Both $\bar{E}(k_{\ell })$ and −Π_𝓁_ have been shifted by 27 days per octave relative to 77 km. −Π_𝓁_ was then phase-shifted an additional 41 days. The reference scale (𝓁_ref_ = 77 km) is an arbitrary reference to show scale self-similarity, as all plots collapse onto the same curve. Thin dashed lines in (D) correspond to individual scales, while thick solid lines show the median across those scales.

Figure 6 (A to C) shows the seasonality of the surface KE spectrum, $\bar{E}(k_{\ell })$, and scale transfer, Π_𝓁_, as a function of time and scale. Similar to (*3*), we show that outside of the Tropics and within the scale range of 40 to 400 km, larger scales reach their seasonal KE maximum later than smaller scales (Fig. 6, A1 to C1). This delay can be regarded as a “spectral advection” signal in which energy moves to larger scales with time, at a timescale of ≈27 days for a twofold increase in 𝓁. The spectral advection speed is illustrated by the dashed black lines in Fig. 6 (A1 and C1). This speed is slightly faster than what was found in (*3*), which was 35 to 45 days per octave for sea surface height derived geostrophic velocities and may be considered as an indication that the geostrophic flow has a higher inertia in responding to the seasonal changes in atmospheric forcing. Within the Tropics, there is no discernible spectral advection signal, and instead all subgyre scales (smaller than 1000 km) reach their seasonal KE maximum at the same time.

From Fig. 6 (A2 to C2), we see that the cascade has spectral advection signal similar to that of KE within the scale band of 60 to 400 km in both hemispheres. Applying the same analysis shows again a period of ≈27 days per octave. While our results are consistent with previous work (*30*, *32*, *37*, *58*) reporting on the mesoscale seasonality, prior focus had often been on seasonality of the spectral power-law scaling in small regions using Fourier analysis. Those studies did not report on the spectral advection shown in Fig. 6 for the global ocean.

#### Time lag between KE spectrum and KE cascade

For any given scale, the energy cascade Π_𝓁_ reaches its seasonal maximum before the KE spectrum $\bar{E}(k_{\ell })$ does, which lags by approximately 41 days. This time lag is highlighted in Fig. 6 in two ways. First, the reference dashed black lines in Fig. 6 (A2 and C2), which show the cascade’s spectral advection speed in the NH and SH, are shifted 41 days earlier than the corresponding lines in Fig. 6 (A1 and C1), which show the spectrum’s spectral advection speed. This result shows that at any scale 𝓁, Π_𝓁_ and $\bar{E}(k_{\ell })$ are 41 days out of phase in their seasonal cycle. Second, in Fig. 6D, we plot the normalized variation of the spectrum and cascade for several scales between 60 and 400 km. Plots of $\bar{E}(k_{\ell })$ (orange) and Π_𝓁_ (blue) are time shifted, with the spectrum plotted as a function of t* = *t* + 27log_2_(𝓁𝓁_ref_) and the cascade as a function of *t*^†^ = t* − 41. Note that 𝓁_ref_ = 77 km is an arbitrary “reference scale.” Changing 𝓁_ref_ merely yields a uniform time shift of all plots in Fig. 6D. With these time shifts, the seasonal signals for $\bar{E}(k_{\ell })$ and Π_𝓁_ (blue and orange in Fig. 6D) collapse on each other for all plotted scales between 60 and 400 km. This result is evidence of self-similar dynamics in the mesoscale range, whereby temporal evolution at different length scales appears identical when properly rescaled. The fact that the cascade peak precedes a peak in the KE spectrum at different scales in Fig. 6 is suggestive of a causal relation between the cascade and variations in the energy content at different mesoscales.

#### Scale locality of the mesoscale cascade

The cascade Π_𝓁_ we are measuring quantifies the rate of KE being transferred across scale 𝓁 and whether it is upscale or downscale. The above findings allow us to make an important conclusion about the length scales at which the cascade is depositing energy. Using two key results from Fig. 6: (i) the spectral advection for both Π_𝓁_ and $\bar{E}(k)$ is 27 days per octave, and (ii) the time lag between Π_𝓁_ and $\bar{E}(k)$ is 41 days; we can infer that Π_𝓁_ at scale 𝓁 is in-phase with $\frac{d}{dt}\bar{E}(k)$, the tendency of the KE spectrum at scale *k*^−1^ = 3.6 𝓁 (see Materials and Methods). This correlation suggests that energy being transferred across scale 𝓁 is primarily deposited at scale ≈4 𝓁, which is in agreement with predictions from 2D turbulence theory (*59*). We caution, however, that unlike in idealized turbulence where the system is closed, in the ocean there are other possible energy sources/sinks besides the cascade that may influence the KE seasonal cycle of scales. While agreement with ideal 2D turbulence theory suggests that the cascade is probably the dominant energy source for this range of scales, ascertaining it requires probing other energy pathways, including potential energy release (*12*, *60*) and eddy damping (*61*–*63*).

### Cascade through the global ocean volume

**Fig. 7.**
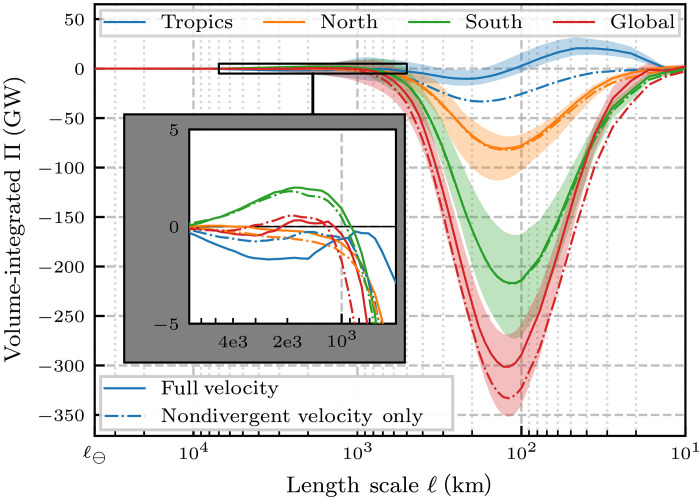
KE cascade. Volume-integrated Π_𝓁_ (gigawatts) over the global ocean, north of 15°N, between 15°S and 15°N, and south of 15°S. Π_𝓁_ < 0 signals an upscale transfer whereas Π_𝓁_ > 0 is downscale. Lines correspond to the time-median value with envelopes showing the full temporal range. 𝓁_⊖_ denotes the equatorial circumference of Earth, ∼40 × 10^3^ km. Note that the horizontal axis, 𝓁, decreases to the right (so that the filtering wave number, 𝓁^−1^, increases to the right). The in-set axis zooms in on the plot portion outlined in the black box, and has the same units as the main axes. Solid lines correspond to results using the full velocity, while dash-dotted lines correspond to results obtained using only the toroidal (laterally nondivergent) flow component. In the in-set axes, 2e3 and 4e3 are shorthand for 2 × 10^3^ and 4 × 10^3^, respectively.

Figure 7 presents the scale transfer of KE (Π_𝓁_) volume-integrated over the global ocean and three regions of interest. Outside of the Tropics, the dominant behavior is a strong upscale cascade (Π_𝓁_ < 0) over the mesoscales. The upscale energy transfer peaks at 𝓁 ≈ 125 km, with a total energy transfer rate of ≈300 GW. This is the first estimate for the global ocean KE cascade. We emphasize that no such estimate is available using either Fourier analysis in regional boxes or structure functions. For comparison, this transfer rate of ≈300 GW is nearly three times larger than a previous global estimate by (*64*) using a temporal Reynolds decomposition, which showed that 110 GW is transferred from the time-mean to the time-varying flow. It is also roughly 40% of the estimated 760 GW that is transferred into the geostrophic surface currents from wind (*24*, *65*) and, like the wind input, is dominated by the southern hemisphere. Excluding the laterally divergent (ageostrophic) flow, which is mostly in the tropics, the upscale mesoscale cascade is slightly stronger at ≈325 GW. Recently, the study in (*63*) showed that while wind forcing drives scales larger than 260 km, it on average removes energy from scales smaller than 260 km through eddy damping. Our result here implies that most of the mesoscale cascade, including the peak, occurs on length scales that are on average damped by the winds.

Figure 7 is simply a meridionally integrated version of Fig. 3. From Fig. 3, it can be seen that 120 km is not the peak transfer scale across all latitudes, owing to variations in the Coriolis parameter. It may be tempting to spatially integrate the scale transfer Π_𝓁(ϕ)_ across different scales 𝓁(ϕ), which vary with latitude ϕ rather than retaining a fixed 𝓁 before spatially integrating as we do in Fig. 7. However, using a filtering kernel of varying width 𝓁(ϕ) to coarse-grain the governing equations does not yield a term Π_𝓁(ϕ)_ in the energy budget because such filtering does not commute with derivatives (see Materials and Methods), which would then render a spatially integrated Π_𝓁(ϕ)_ of little dynamical meaning. An advantage of our scale decomposition is that it guarantees energy conservation (*42*), which follows from our generalized convolution commuting with spatial derivatives on the sphere, thereby preserving scale-dependent symmetries (*66*).

#### Southern hemisphere dominance of the mesoscale cascade

The majority of the upscale transfer occurs south of the tropics (Fig. 7), with a peak SH cascade value approximately 2.7 times larger than the NH value. Because the total water volume of the SH (≈45% of the global ocean volume) is roughly 1.7 times larger than the NH (≈26% of the ocean), the discrepancy in energy cascade cannot be solely attributed to increased volume and instead indicates higher mean Π_𝓁_ density. Because the net energy cascade in the Tropics is an order of magnitude smaller, the global mesoscale energy cascade roughly partitions as 73% occurring in SH and 27% in NH.

#### Scale transfer in the tropics

The tropics (15°S to 15°N) present a qualitatively different Π signature than the extratropics (Fig. 7). We find that the volume-integrated Π_𝓁_ is an order of magnitude smaller than that of the extratropics, despite having a volume slightly larger than NH at ≈29% of the total ocean volume. The tropical KE transfer is downscale for length scales smaller than ∼100 km. Excluding the divergent (ageostrophic) flow, the transfer in the tropics is upscale. This result is consistent with the notion that flow in the tropics is less constrained by 2D-like geostrophic dynamics and is more similar to 3D turbulence, which exhibits a downscale cascade (*12*, *17*).

#### Gyre-scale KE scale transfer

The in-set axes of Fig. 7 focuses on the range of 500 to 7 × 10^3^ km. Being surface-trapped and exhibiting alternating upscale and downscale transfer with latitude, the gyre-scale signal has a much smaller volume-integrated magnitude [𝒪(1) GW] relative to the mesoscale cascade, which is mostly upscale and penetrates the entire water column (Fig. 3, E and F). Despite being much weaker on a global spatial average, the gyre-scale transfer is spatiotemporally persistent and directly affects the globally coherent oceanic circulation patterns. South of the tropics, these scales exhibit a net downscale transfer even when removing the Ekman flow component (nondivergent green plot in Fig. 7, inset). We have seen from the maps in Fig. 2E that such downscale transfer appears in regions of strong shear, most prominently between the eastward ACC and the westward Agulhas current (see also Fig. 3B). This result presents another north/south asymmetry, as the NH exhibits a net upscale transfer over the gyre scales (orange plot in Fig. 7, inset). While there is a downscale transfer in the flanks of the Gulf Stream and Kuroshio currents (red in Fig. 2E), the shear is not sufficiently strong to dominate over the upscale transfer in the currents’ cores. Flow in the tropics exhibits an upscale transfer on average (blue in Fig. 7, inset), primarily due to the equatorial Ekman divergence at gyre scales (see maps in Fig. 2). Yet, even if the divergent component is excluded, an upscale KE transfer is still present at those scales (dashed blue in Fig. 7, inset).

## DISCUSSION

Using a coarse-graining approach developed for analyzing scale dynamics on the sphere (*66*), and implementing it in an efficient parallel code, FlowSieve (*67*), we were able to chart the first global maps of the KE cascade in the ocean. Scale transfer of oceanic KE across gyre scales revealed a prominent imprint of the atmosphere’s Hadley, Ferrel, and polar circulation cells through five latitudinal bands of alternating upscale and downscale KE transfer. We provided evidence that such gyre-scale transfer occurs because of KE exchange with the mesoscales and proposed a theory to explain the transfer mechanism. Our analysis also found that the atmosphere’s ITCZ produces a narrow latitudinal band of intense downscale transfer, which exhibits a seasonal meridional shift following the ITCZ.

An important accomplishment of this work is quantifying the Ekman transport’s role in the KE scale transfer directly from oceanic velocity. Traditionally, Ekman transport had been inferred only indirectly from wind stress. This was key to demonstrating the existence of energy exchange between gyre scales and mesoscales, which can be understood using a piston-pressure framework that is unorthodox in physical oceanography. This transfer is not part of the standard theory of gyre circulation, either Stommel’s or Fofonoff’s (*12*). The existence of such transfer implies that the mesoscale eddies play an important role in the momentum of gyre scale by exerting an effective pressure (normal stress). This was not recognized before, probably because typical analysis of the gyre circulation relies on Sverdrup-like relations based on vorticity balance (*12*), which, being the curl of momentum balance, misses the effective mesoscale pressure process underlying the gyre-scales–mesoscales transfer.

Probing the scale transfer of KE across scales ranging from 10 to 40 × 10^3^ km, we were also able to provide the first estimate of the global oceanic cascade, which has a peak upscale transfer rate of 300 GW. This is a substantial fraction (≈13) of the wind power driving the oceanic general circulation (*24*, *65*) and constitutes a previously unquantified source in the global energy budget of the ocean’s mesoscales between 100 and 500 km in size (*22*). The comprehensive scale analysis undertaken in this work, spanning 3.5 decades in length scale, 4 years of daily averaged velocity, and covering the global ocean volume, including 50 depth levels, has not been performed before. In fig. S7 in the SM, we also present supporting analysis from 9 years of observational data from satellite altimetry.

We showed that both the mesoscale upscale KE cascade and KE spectrum display a type of scale self-similarity going to larger scales until reaching ≈500 to 10^3^ km. Our analysis indicates that the mesoscale cascade and spectrum follow the same self-similar seasonal cycle (27 days per octave) but are 40 days out of phase, which suggests that the KE transferred across any mesoscale 𝓁 is primarily deposited at a scale four times larger. Evolution of scales larger than 10^3^ km starts being affected by gyre-scale strain, which is spatially inhomogeneous, shaped by domain geometry and wind forcing. We found that the upscale mesoscale cascade is dampened substantially at scales of only a few hundred kilometers (Fig. 7). This damping may be due to generation of Rossby waves at scales larger than the Rhines scale 𝒪(100) km, which decorrelates the nonlinear interactions in the meridional direction (*68*, *69*). However, such decorrelations do not inhibit the upscale cascade from generating larger scales in the east-west direction, for which we find no evidence from our analysis. Moreover, the length scale at which the mesoscale cascade is arrested decreases poleward (Fig. 3) unlike the Rhines scale, which does not exhibit substantial variations with latitude (see fig. S4 and (*70*)]. These observations reinforce previous studies (*31*, *71*, *72*) indicating that Rossby wave generation does not adequately explain the upscale cascade’s arrest. It is likely that the cascade is attenuated at a few hundred kilometers because of frictional processes that act at these scales in a more isotropic fashion, including eddy damping by wind (*61*–*63*) and bottom drag (*25*). Determining the relative contribution of these mechanisms to the cascade’s “arrest” is left to future work. Our findings are consistent with the presence of KE sources and sinks over a wide range of scales in the realistic ocean, unlike in idealized turbulence theories (*17*) where energy is transferred conservatively across scales within the so-called inertial range.

Concerning one motivation in the Introduction on the possibility of energy transfer between mesoscales and the larger coherent circulation leading to intrinsic ocean and climate variability, our demonstration of the existence of such transfer should stimulate further scrutiny into the matter. Even if the gyre-scales–mesoscales exchange is a mere fraction of a percent of the 300-GW mesoscale upscale transfer, it can play a meaningful role in climate dynamics. This is because the gyre-scale–mesoscale KE transfers, while being weaker than those due to self-interactions among the geostrophic mesoscale eddies, probably have a disproportionate effect on the global circulation patterns and climate due to the gyre scales’ coherence in space and persistence in time. A rudimentary estimate shows that a net increase of a mere 0.3 GW (0.1% of the 300 GW) in KE transfer to the gyre scales of a major circulation pattern, either via amplified upscale transfer or via attenuated downscale transfer, can be momentous. For example, in the case of the ACC’s ≈10 cm/s gyre-scale (>10^3^ km) speed (*42*), such a variation would result in a 10% speed increase over 10 years and over a volume of ≈10^8^ km^3^ assuming that energy sinks remain unchanged. This would be a considerable speed-up, 𝒪(10)× observed acceleration (*73*). Such an estimate is only intended to highlight how minuscule variations in scale transfer can have a potentially meaningful impact on gyre-scale circulation, and the importance of advancing rigorous scale transfer analysis to complement established approaches such as mean-eddy interactions theory and investigations of eddy saturation, standing meanders and bottom topography, among many others (*40*). Our work lends support to recent results (*9*) showing that the strengthening of mesoscale ocean eddies leads to a strengthening of climate variability. Above results also highlight the gyre circulation’s departure from linear balance and the potentially important role of effective pressure (normal stress) exerted by the mesoscales, which penetrate the entire water column (Fig. 1), in coupling the ocean surface to the deeper circulation. By quantifying the transfer of energy across scales, we view our work as laying a promising framework for tackling the problem of multiscale coupling within the climate system.

## MATERIALS AND METHODS

### NEMO dataset

The dataset analyzed in this work is publicly available from Copernicus Marine Service (CMEMS). The specific product identifier of the NEMO dataset used here is “GLOBAL_ MULTIYEAR_PHY_001_030.” (DOI: 10.48670/moi-00021). The ocean reanalysis model has 112° horizontal grid spacing with 50 vertical levels spanning 5.5 km of depth, with 22 of the vertical levels in the top 100 m. Because of the constant land mask in Antarctica, the NEMO dataset does not extend to 90°S. We extend the dataset to the southern pole by extending the land mask (i.e., zero velocity). This is done so that filtering kernels intersecting Antarctica are not erroneously truncated.

#### Surface results time series

For results considering only the ocean surface [i.e., Figs. 2, 3 (A to D), and 6], we analyze the daily mean ocean velocities at daily resolution for the 4 years spanning 2015–2018.

#### Depth results time series

Because of the increased computational cost of analyzing full-depth data, when considering depth-dependent results (i.e., Figs. 1, 7, and 3, E and F) the time series of full-depth results only includes the first day of each month, as opposed to full daily resolution. That is, 1 January, 1 February, ..., 1 December, for the 4 years spanning 2015–2018.

### AVISO dataset

The AVISO dataset analyzed in the Supplementary Materials is also available through CMEMS under the product identifier “SEALEVEL_GLO_PHY_L4_MY_008_047” (DOI: 10.48670/moi-00145). This dataset provides daily mean velocities, gridded at 14° resolution with global coverage.

### Rossby number

Figure 3 (A to D) and the resulting discussion use the Rossby number (Ro), and specifically when Ro = 0.1. In this work, the Rossby number is calculated as Ro = *U*/(*f*𝓁), where 𝓁 is the filter scale, *f* is the Coriolis parameter, calculated at each latitude, and $U=\sqrt{2\rho^{-1}{\mathrm{KE}}^{>\ell }}$ is the root-mean-squared velocity containing only scales larger than 𝓁 (*37*). This approach results in a Rossby number that is a function of both scale and latitude.

### Coarse-graining

Coarse-graining in simple terms is a convolution between a scalar function being filtered, *F*, and the filtering kernel *G*. This can be viewed as a spatially weighted average, performed in a careful manner. For a chosen length scale 𝓁, given in metres, we define the coarse-grained (i.e., low-pass filtered) scalar function ${\bar{F}}_{\ell }$ as

$${\bar{F}}_{\ell }(\vec{x})=\int_{\text{Ω}}\;F(\vec{y})G_{\ell }[\gamma (\vec{x},\vec{y})]dS(\vec{y})$$ (1)

where *G*_𝓁_ is the filtering kernel, $dS(\vec{y})$ is the area measure on the sphere, Ω is the entire spherical shell, and $\gamma (\vec{x},\vec{y})$ is geodesic distance between the points $\vec{x}$ and $\vec{y}$

$$\gamma (\vec{x},\vec{y})=R_{E}\arccos\;[\sin\phi_{x}\sin\phi_{y}+\cos\phi_{x}\cos\phi_{y}\cos(\lambda_{x}-\lambda_{y})]$$ (2)

with *R*_E_ = 6371 km for Earth’s radius and ϕ, λ are the latitude and longitude of $\vec{x}$ and $\vec{y}$.

There are many possible choices for filtering kernel, but desirable properties are *G*(γ) ≥ 0 for all γ and *G*(γ) → 0 for γ > 𝓁/2. In this work, we use the graded top-hat filter of (*3*), such that the kernel with length scale 𝓁 is given by

$$G_{\ell }(\gamma )=\frac{A}{2}\{1-\tanh\left[10\left(\frac{\gamma }{\ell /2}-1\right)\right]\}$$ (3)

In Eq. 3, *A* is a normalization factor, evaluated numerically, to ensure that *G*_𝓁_ integrates to unity over the full sphere. The careful mathematical formulation (*66*) and numerical implementation (*67*) of coarse graining allows us to preserve flow symmetries at different scales because the operation commutes with derivatives, which enables us to extract meaningful flow diagnostics (*33*). Ensuring that convolutions and spatial derivatives commute is mathematically nontrivial on the sphere and relies on generalizing the convolution operation (*66*). The difficulties with commutation are due to curvature, irrespective of any discretization on numerical grids.

#### Land treatment

Following (*3*), we treat land as zero velocity water for the purpose of coarse graining, which is consistent with the boundary conditions. However, whereas (*3*) applied this treatment in the filtering step (i.e., calculating Eq. 1), in this work it is applied during the Helmholtz projection step, which automatically respects the oceanic flow’s boundary conditions. When coarse-graining at a scale 𝓁, the precise boundary between land and ocean becomes blurred at that scale and its precise location becomes less certain. The coarse-grained velocity, ${\bar{u}}_{\ell }$, is allowed to be nonzero within a distance 𝓁/2 beyond the continental boundary over land (*42*). Otherwise, requiring ${\bar{u}}_{\ell }$ to vanish over land entails deforming the kernel *G*_𝓁_ in Eq. 3, which breaks the flow symmetries at different scales, i.e., coarse graining would no longer commute with differential operators (*66*). Forfeiting exact spatial localization to gain scale information is theoretically inevitable because of the uncertainty principle (*3*, *34*).

#### Reference density

When calculating KE^>𝓁^ and Π_𝓁_, we use of the reference density ρ = ρ_0_ = 1025 kg m^−3^, as per the Boussinesq approximation used by the NEMO simulation.

#### FlowSieve

The software package used to perform the coarse-graining calculations is FlowSieve (*67*), which was developed by the authors. Source code is available at github.com/husseinaluie/FlowSieve, with documentation at flowsieve.readthedocs.io.

### Diagnostic quantities: Filtering spectrum

Following (*34*), we define the coarse KE per volume as ${\mathrm{KE}}^{>\ell }=0.5\rho \;{\bar{u}}_{i}{\bar{u}}_{i}$ (i.e., the KE of the coarse-grained velocity) and, subsequently, the filtering spectrum as its *k*_𝓁_ derivative

$$\bar{E}(k_{\ell })=\frac{d}{dk_{\ell }}\;{\mathrm{KE}}^{>\ell }\;(\text{Filtering spectrum})$$ (4)

where *k*_𝓁_ = 𝓁^−1^. The filtering spectrum is analogous to the traditional Fourier power spectrum when the latter is valid [c.f. figure 4 of (*3*)]. For a positive semidefinite kernel such as the one used here, the steepest resolvable spectral slope is −3 (*34*). However, because we do not measure slopes as steep as −3 (c.f. Fig. 1), this limitation is not a concern for our applications.

### Diagnostic quantities: KE scale transfer

KE scale transfer arising from the nonlinear dynamics is obtained from coarse-graining (*74*–*78*) the flow equations as

$$\prod_{\ell }:=-\frac{\rho }{2}({\bar{u}}_{i;j}+{\bar{u}}_{j;i})T^{ij}$$ (5)

where repeated indices denote summation, indices after semicolon (;) denote (covariant) differentiation, and 𝒯*^ij^* are (contravariant) components of the subscale stress (per unit mass) tensor ${\bar{u\;u}}_{\ell }-{\bar{u}}_{\ell }\;{\bar{u}}_{\ell }$ (*33*). For ocean flows, the term in Eq. 5 arises from applying the generalized version [see (*66*)] of the convolution in Eq. 1 to the equation of fluid momentum (per unit mass) on a sphere

$$\frac{\partial }{\partial t}{\bar{u}}_{\ell }+\nabla \cdot ({\bar{u}}_{\ell }\;{\bar{u}}_{\ell })+\cdots =-\nabla \cdot ({\bar{u\;u}}_{\ell }-{\bar{u}}_{\ell }\;{\bar{u}}_{\ell })+\cdots$$ (6)

where   ∇ · () is (covariant) divergence. Equation 6, which governs the flow at scales >𝓁, is only possible to derive if the convolution commutes with spatial derivatives (*33*, *66*). Commutation is violated, for example, if filtering is performed by using a spatially varying kernel or by using facile averaging of tensors on the sphere. Taking the inner product of Eq. 6 with $\rho \bar{u}$ yields the coarse KE budget

$$\partial_{t}(\rho {|{\bar{u}}_{\ell }|}^{2}/2)+\cdots =-\prod_{\ell }+\cdots$$ (7)

in which Π_𝓁_ appears as a sink that is Galilean invariant and scale local under certain conditions (*77*). Π_𝓁_ is signed so that positive values indicate energy transfer from scales larger than 𝓁 to scales smaller than 𝓁 (*79*). Where the KE scale transfer can be reasonably argued to be scale-local, we refer to Π_𝓁_ as the KE “cascade.”

Despite using isotropic kernels for coarse graining (Eq. 3), Π_𝓁_ can still detect anisotropic energy transfer (e.g., due to the Ekman flow) but it, alone, cannot inform us about the direction along which such transfer occurs. To highlight the role of gyre-scale divergence (e.g., due to Ekman transport) in scale transfer, we can expand Eq. 5 to obtain

$$\prod_{\ell }=-\frac{\rho }{2}[{\bar{u}}_{\lambda ;\lambda }T^{\lambda \lambda }+{\bar{u}}_{\phi ;\phi }T^{\phi \phi }+\cdots ]$$ (8)

From Eq. 8, a meridionally convergent flow, i.e., ${\bar{u}}_{\phi ;\phi }<0$, yields a positive contribution to Π_𝓁_ (i.e., downscale KE transfer) because 𝒯^ϕϕ^ is positive semidefinite and represents a portion of the fine KE, at scales <𝓁 (*34*, *44*). Π_𝓁_ can be written as Π(**u**, **u**, **u**) to highlight its dependence on three velocity modes (*77*). The first of these modes, Π(**u**, ·, ·) contributes to the strain in Eq. 5, while the second and third modes contribute to the subscale stress ${\bar{u\;u}}_{\ell }-{\bar{u}}_{\ell }\;{\bar{u}}_{\ell }$ (*77*). Given that the lateral velocity **u** can be decomposed into a laterally nondivergent (T, for toroidal) and laterally divergent (D) components (*66*), Π can be decomposed exactly into a sum of eight terms: Π(*T*, *T*, *T*), Π(*T*, *T*, *D*), Π(*T*, *D*, *T*), Π(*T*, *D*, *D*), Π(*D*, *T*, *T*), Π(*D*, *T*, *D*), Π(*D*, *D*, *T*), and Π(*D*, *D*, *D*). These terms can represent a different mechanism for KE scale transfer. For example, because the nondivergent flow (*T*) is predominantly geostrophic the Π(*T*, *T*, *T*) term expresses transfer due to self-interactions among the geostrophic mesoscale eddies, which generally yield an upscale cascade. By subtracting Π(*T*, *T*, *T*) from Π, we are left with the scale transfer due to interactions involving the divergent flow (e.g., Ekman flow and other unbalanced motions). This is similar, at least in spirit, to the analysis in (*44*) at the submesoscales.

#### Radial/vertical velocity

In computing both the KE spectrum and scale transfer, only the zonal and meridional velocity components are considered. For hydrostatic flows at scales considered here, the lateral flow makes the overwhelming KE contribution. We conducted identical analysis that included radial/vertical velocities diagnosed using flow incompressibility, and found that including the radial velocity *u_r_* has a negligible impact on both diagnostics across all scales analyzed here.

#### Ekman velocity

Calculations of Π_𝓁_ and $\bar{E}(k_{\ell })$ relied on the full velocity from the NEMO reanalysis. However, to interpret those results, we sometimes appealed to the Ekman velocity, which is defined as (*55*)

$$u_{\lambda }^{E}=\frac{1}{\rho \cdot H^{E}}\frac{f(\phi )}{f{\left(\phi \right)}^{2}+\varepsilon^{2}}\;\tau_{\phi }\;(\mathrm{zonal})$$ (9)

$$u_{\phi }^{E}=-\frac{1}{\rho \cdot H^{E}}\frac{f(\phi )}{f{\left(\phi \right)}^{2}+\varepsilon^{2}}\;\tau_{\lambda }\;(\mathrm{meridional})$$ (10)

where ρ = ρ_0_ is the reference density, and *H^E^* = 50 m is taken to be the gyre-scale Ekman layer depth motivated by Fig. 3F, although taking *H^E^* to be the seasonally varying mixed layer depth (fig. S2 in the SM) yields the same conclusions. *f*(ϕ) is the local Coriolis parameter, ɛ = 3.2 × 10^−6^s^−1^ ≈ *f*(1.25°) is the mechanical damping rate (*55*), and τ_λ_ is the zonal relative wind stress provided by the NEMO reanalysis data, which follows a bulk formulation

$$\left(\tau_{\lambda },\tau_{\phi })=\rho_{\mathrm{air}}C_{D}|{\vec{u}}_{\mathrm{air}}-{\vec{u}}_{\mathrm{ocean}}|({\vec{u}}_{\mathrm{air}}-{\vec{u}}_{\mathrm{ocean}}\right)$$ (11)

The density of air (ρ_air_), drag coefficient (*C*_D_), and 10 m air velocity $\left({\vec{u}}_{\mathrm{air}}\right)$ used in the NEMO ocean reanalysis model are obtained from ERA5 (ECMWF [European Centre for Medium-Range Weather Forecasts] ReAnalysis v5) atmospheric reanalysis [“*ERA5 hourly data on single levels from 1940 to present*”. Copernicus Climate Change Service (C3S) Climate Data Store (CDS), DOI: 10.24381/cds.adbb2d47]. Where necessary, linear interpolation on latitude-longitude grids was applied to bring the ERA5 data fields onto the same grid as the NEMO ocean data $\left({\vec{u}}_{\mathrm{ocean}}\right)$.

#### Time lag and scale locality of the mesoscale cascade

For the seasonality at each 𝓁, both −Π(𝓁, *t*) and $\bar{E}(\ell ,t)$ have a period of 365 days but $\bar{E}(\ell ,t)$ is phase shifted 41 days after −Π(𝓁, *t*). Therefore, the KE spectrum tendency, $\frac{d}{dt}\bar{E}(\ell ,t)$, is phase shifted −41 + 365/4 = 50 days before −Π(𝓁, *t*). Noting that the cycle for each of −Π(2𝓁, *t*) and $\frac{d}{dt}\bar{E}(2\ell ,t)$ at scale 2𝓁 is phase shifted 27 days later relative to that at scale 𝓁, we have that the upscale cascade, −Π(𝓁, *t*), is in-phase with $\frac{d}{dt}\bar{E}(3.6\ell ,t)$ at length scale 3.6𝓁 ≈ 4𝓁.

#### Horizontal, vertical, and temporal averages and integrals

The diagnostics variables are computed at all points in space and time for the entire dataset of consideration. That is, Π_𝓁_ = Π_𝓁_(*t*, *z*, ϕ, λ), for time *t*, depth *z*, latitude ϕ, and longitude λ.

1) Horizontal averages (such as Fig. 1) are spatial integrals normalized by the spatial area, with appropriate weighting by the cell area

$$\mathrm{HorizAvg}(F)(t,z,\text{Ω})=\frac{\int_{(\phi ,\lambda )\in \text{Ω}}F(t,z,\phi ,\lambda )dA}{|\text{Ω}|}$$ (12)

where Ω is the spatial region of interest and

$$|\text{Ω}|=\int_{(\phi ,\lambda )\in \text{Ω}}\mathrm{IsWater}(\phi ,\lambda ,z)dA$$

is the water area [i.e., IsWater(ϕ, λ, *z*) = 1 if (ϕ, λ, *z*) is a water cell and 0 otherwise]. Horizontal averages are then functions of time, depth, and the choice of region. Horizontal integrals remove the normalization factor ∣Ω∣^−1^.

2) Zonal averages (such as Fig. 3, A to F), are computed along lines of constant latitude. As with horizontal averages, zonal averages are normalized by water area at each latitude.

3) Depth integrals account for the depth-varying vertical thickness of cell grids by treating z levels as cell bottoms, and extending the top cell (depth of ∼0.5 m) to the surface. Vertical thicknesses vary monotonically from ∼0.5 to ∼450 m.

4) Time averages (both means and medians) are computed in the standard way, because we have uniform time sampling. In the case of seasonal averages (e.g., Fig. 7), the time series is partitioned on the basis of the month of the year, with the mean/median of each partition computed separately.

### Helmholtz decomposition

Unlike the analysis in (*3*) using geostrophic velocity, the results presented here use the full horizontal model velocity, which contains both rotational and horizontally divergent components. This generality renders coarse-graining the velocity field in a manner that commutes with spatial derivatives more complicated, involving the so-called Edmonds transformation (*66*). A solution we use here is to first perform a Helmholtz decomposition of the velocity field and obtain coarse velocities from the coarse-grained Helmholtz scalars, which is equivalent to performing the Edmonds transformation (*66*). Specifically, if

$$\vec{u}=\left[\begin{array}{c} u_{\lambda }\\ u_{\phi } \end{array}\right]=\left[\begin{array}{cc} -\frac{\partial }{\partial \phi } & \sec(\phi )\frac{\partial }{\partial \lambda }\\ \sec(\phi )\frac{\partial }{\partial \lambda } & \frac{\partial }{\partial \phi } \end{array}\right]\left[\begin{array}{c} \text{Ψ}\\ \text{Φ} \end{array}\right]$$ (13)

where λ, ϕ are the longitude and latitude, *u*_λ_, *u*_ϕ_ are the zonal and meridional velocities, and Ψ, Φ are the Helmholtz scalars, then

$$\bar{\vec{u}}=\left[\begin{array}{cc} -\frac{\partial }{\partial \phi } & \sec(\phi )\frac{\partial }{\partial \lambda }\\ \sec(\phi )\frac{\partial }{\partial \lambda } & \frac{\partial }{\partial \phi } \end{array}\right]\left[\begin{array}{c} \bar{\text{Ψ}}\\ \bar{\text{Φ}} \end{array}\right]$$ (14)

where $\bar{\text{Ψ}},\bar{\text{Φ}}$ are computed by coarse-graining each field as a scalar (*66*, *67*). Computational details of the Helmholtz decomposition, which uses the ALGLIB package (*80*), can be found in the Supplemental Materials.
