# Supplementary material for: Global cascade of kinetic energy in the ocean and the atmospheric imprint
Source: Sci Adv. 2023 Dec 20;9(51):eadi7420. doi: 10.1126/sciadv.adi7420 (PMC10732517; doi:10.1126/sciadv.adi7420)
Supplement: Supplementary file 1 — Figs. S1 to S8 [file sciadv.adi7420_sm.pdf]

Science Advances
AAAS

Supplementary Materials for

Global cascade of kinetic energy in the ocean and the atmospheric imprint

Benjamin A. Storer *et al.*

Corresponding author: Hussein Aluie, hussein@rochester.edu

Sci. Adv. **9**, eadi7420 (2023)
DOI: 10.1126/sciadv.adi7420

This PDF file includes:

Figs. S1 to S8

POWER SPECTRA AT VARIOUS DEPTH

Figure S1 is supplemental to Figure 1, and shows the power spectra of selected depths, along with the spectrum of the depth-average flow (solid black lines) and the depth-average of the power spectra (dashed black lines).

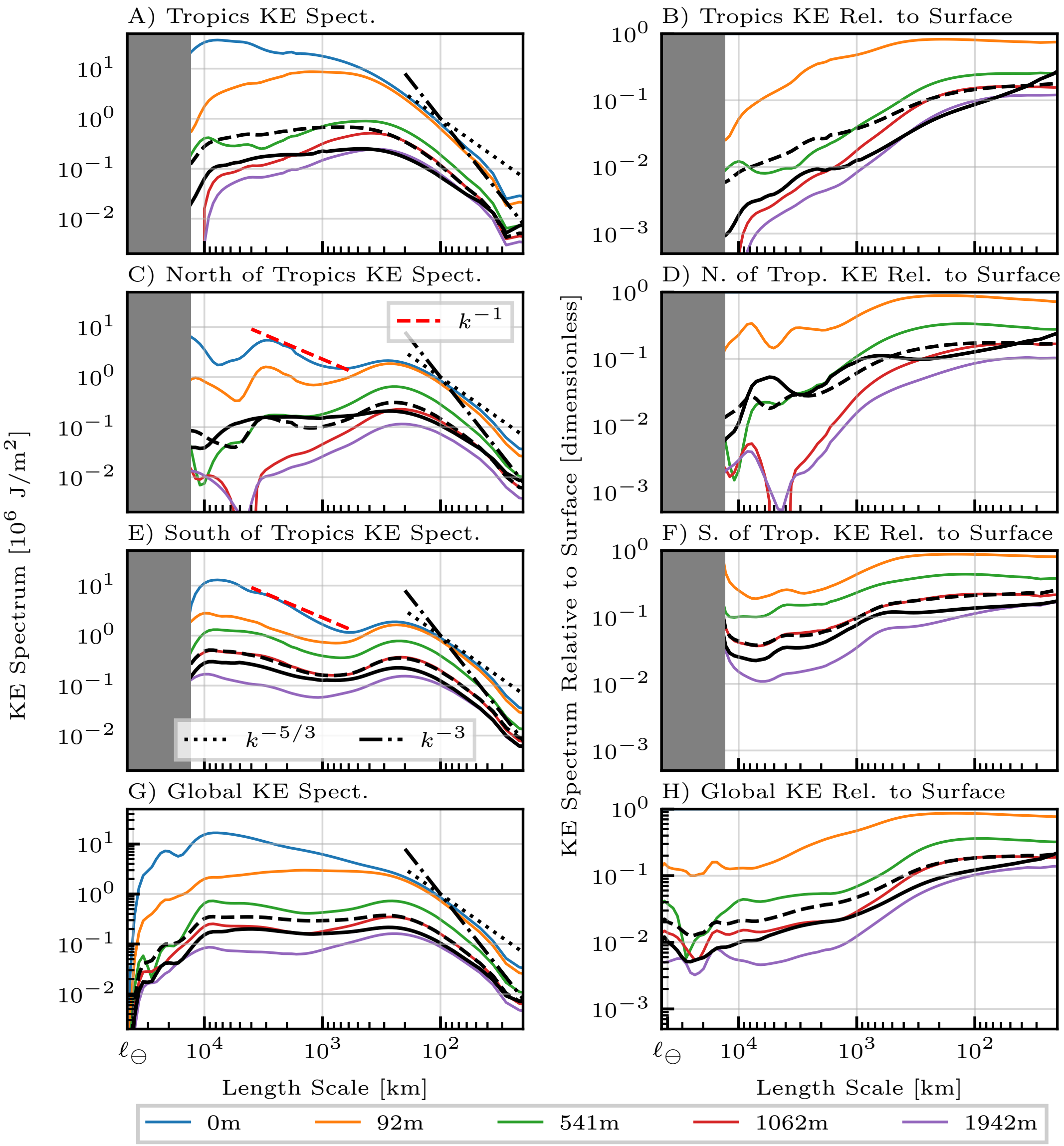

Figure S1: KE Spectra at Selected Depths [A,C,E,G] Similar structure to Figure 1, but with spectra plotted only for selected depths [see in-set legend]. [B,D,F,H] are again similarly structured, but now showing the KE spectrum divided by the surface spectrum. [Thick solid black lines] show spectra of the depth-averaged flow, while [thick dashed black lines] show the depth-averaged spectra.

HELMHOLTZ COMPUTATIONAL FRAMEWORK

The Helmholtz system to be solved is given by eq. S-1. Nominally, those two equations alone would be sufficient to determine the Helmholtz scalars.

$$\begin{bmatrix} \nabla^2 & 0 \\ 0 & \nabla^2 \end{bmatrix} \begin{bmatrix} \\ \Phi \end{bmatrix} = \begin{bmatrix} \zeta \\ \delta \end{bmatrix} \tag{S-1}$$

However, because the second derivatives both share even symmetry, spurious grid-scale noise can arise. To resolve this, two additional rows are added, yielding eq. S-2. The first two rows of eq. S-2 define the relationship between the velocities, u_λ, u_ϕ, and the Helmholtz scalars, Ψ, Φ (i.e. eq. 13), and the second two impose that the vorticity ζ and divergence δ be wholly described by Ψ and Φ respectively. Using both the upper and lower halves of the least-squares problem reduces computationally spurious noise by including both first derivatives, which have odd-symmetry stencils, and second order derivatives, which have even-symmetry stencils. The scaling factor α allows tuning between the upper and lower halves of the projection operator, while the Laplacian is given by

$$\nabla^2 := (\cos\phi)^{-2}\partial^2_{\lambda\lambda} + \partial^2_{\phi\phi} - \tan(\phi)\partial_\phi.$$

$$\begin{bmatrix} -\partial_\phi & \sec(\phi)\partial_\lambda \\ \sec(\phi)\partial_\lambda & \partial_\phi \\ \alpha\nabla^2 & 0 \\ 0 & \alpha\nabla^2 \end{bmatrix} \begin{bmatrix} \\ \Phi \end{bmatrix} = \begin{bmatrix} u_\lambda \\ u_\phi \\ \alpha\zeta \\ \alpha\delta \end{bmatrix} \tag{S-2}$$

The Helmholtz scalars are computed using an iterative least-squares solver. Specifically, eq. S-2 is converted into a sparse matrix problem by replacing the differential operators with matrix operators using finite difference approximations. The results presented in this work used a fourth-order finite difference scheme. The solve step is performed using the sparse least-squares solver provided in ALGLIB [80]: `linlsqrsolvesparse`, a matrix-free iterative solver. Convergence of the iterative solver is improved by first downsampling the velocities u_λ, u_ϕ onto coarser grids, solving for the Helmholtz scalars on the coarse grid, and providing the coarse solution as an initial guess for the higher resolution solve.

BOUNDARY CONDITIONS

Our treatment of land cells as zero-velocity water cells automatically imposes the boundary conditions required for solving eq. S-1 (or eq. S-2), which we do over the entire spherical domain.

BUILDING THE LEAST-SQUARES MATRICES

The entries in eq. S-2 are block matrices. That is, each entry itself represents a matrix constructed using the standard Kronecker product method, outlined here for the purpose of completeness. Suppose the grid has N_ϕ, N_λ points in latitude and longitude. Then let I^ϕ, I^λ be the $N_\phi \times N_\phi$ and $N_\lambda \times N_\lambda$ identity matrices. Let D^ϕ, D^λ be the finite difference first-derivative matrices on the ϕ and λ grids (of size $N_\phi \times N_\phi$ and $N_\lambda \times N_\lambda$, respectively). The entries of eq. S-2 are then built using Kronecker products: $\partial_\phi = \mathrm{Kron}(D^\phi, I^\lambda)$, $\partial_\lambda = \mathrm{Kron}(I^\phi, D^\lambda)$, etc., so that each sub-array is of size $N_\phi N_\lambda \times N_\phi N_\lambda$. The final least-squares problem is a matrix of size $4N_\phi N_\lambda \times 2N_\phi N_\lambda$, with the solution array a vector of length $2N_\phi N_\lambda$. Since we use fourth-order finite difference derivatives, the least squares matrix is very sparse.

MIXED LAYER DEPTH

Figure S2 presents the monthly-mean Mixed Layer Depth (MLD) as a function of latitude. The lines illustrate how the MLD can increase 3–4 times during the local winter compared to the local summer.

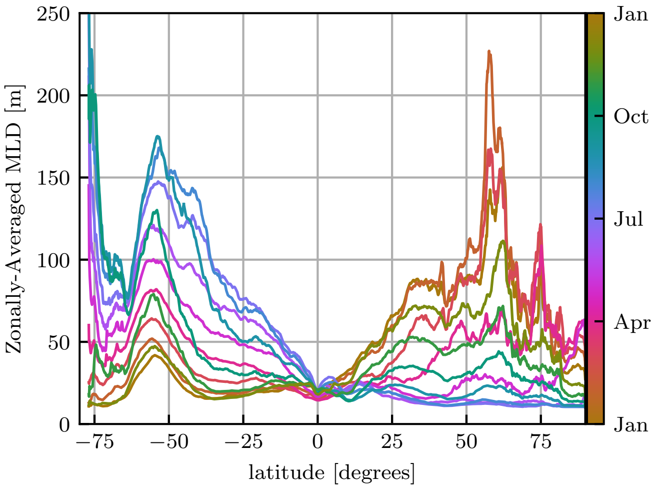

Figure S2: Mixed Layer Depth The monthly-mean zonally-averaged mixed layer depth (MLD). It is provided in the NEMO dataset and corresponds to 2018.

ANNOTATED ZONALLY AVERAGED Π

Figure S3 provides an annotated version of Figure 3C-D, with annotations showing the **[green circles]** show the mesoscale inverse cascade,**[blue rounded boxes]** show the "blue tongue", **[yellow oblong shapes]** show that "red branches", and **[purple rounded boxes]** show the Ekman pattern from the atmospheric cells. The annotations are purely qualitative and for the purpose of illustration.

RHINES SCALE

With coarse-graining, we can define the Rhines scale [68, 69] as a function of latitude (ϕ) as the solution to the following implicit equation,

$$\ell_{\text{Rhines}}(\phi) = 2\pi\sqrt{u_{\text{rms}}(\ell_{\text{Rhines}}, \phi)/\beta(\phi)}, \tag{S-3}$$

where $u_{\text{rms}}(\ell, \phi) = \sqrt{2\rho^{-1}\text{KE}^{>\ell}(\phi)}$ is the rms-velocity of all scales larger than ℓ at latitude ϕ. To solve eq. (S-3), we evaluate the right-hand-side (RHS) for each ϕ over the entire range of scales ℓ and find where the RHS equals ℓ. From the NEMO data, we find that $\ell_{\text{Rhines}}(\phi) \approx 500 \pm 100$ km, without any obvious dependence on latitude ϕ. This result is consistent with previous estimates of the Rhines scale to be $\mathcal{O}(100)$ km without a clear variation with latitude (Fig. 25 in [70]). However, Figure 3 shows that the length-scale at which the mesoscale upscale cascade is arrested decreases poleward, which is not reflected in the Rhines scale. This result suggests that the generation of Rossby waves, also known as the β-effect [68, 69], is probably not the main mechanism by which the mesoscale cascade is arrested. Figure S4 reproduces Figure 3 with an additional line showing the Rhines scale as a function of latitude. The Rhines scale is found to be mostly in the interval $[400\text{ km}, 600\text{ km}]$ and, unlike the deformation radius or scale of peak mesoscale cascade, is broadly constant across latitudes. The Rhines scale's poor correlation with the arrest scale is unrelated to limited resolution of the dataset at high latitudes. Indeed, both the peak cascade scale (orange lines in Figure S4) and transition scale (black lines in Figure S4) decrease at higher latitudes, as expected.

MAPS OF SEASONAL ENERGY TRANSFER

Figure S5 presents the analogue of Figure 2A-D, but showing all four seasons.

MAPS OF TOROIDAL ENERGY TRANSFER

Figure S6 presents the analogue of Figure 2A-D, but for the energy scale-transfer arising solely from the laterally non-divergent flow component.

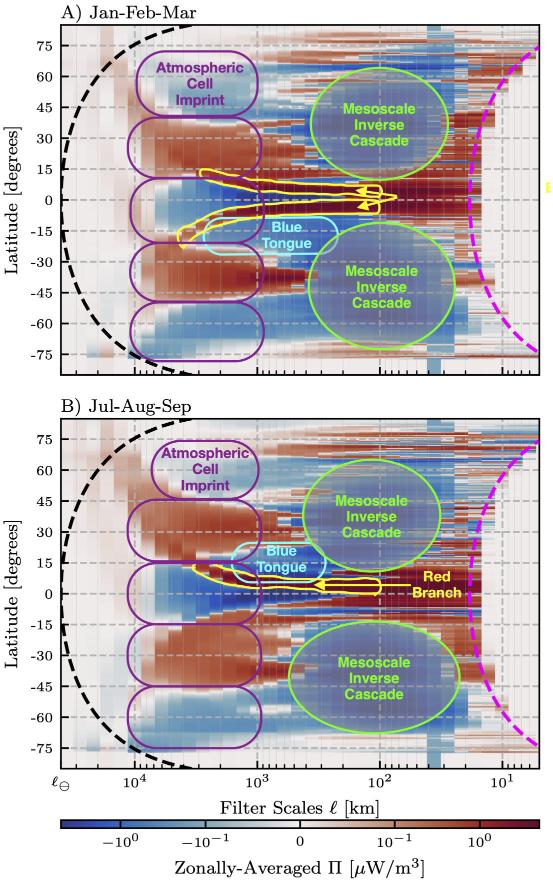

Figure S3: Annotated version of zonal mean Π Reproduction of Figure 3C-D with additional annotations overlain. The annotations are: [green circles] show the mesoscale inverse cascade, [blue rounded boxes] show the "blue tongue," [yellow oblong shapes] show that "red branches", and [purple rounded boxes] show the Ekman pattern from the atmospheric cells.

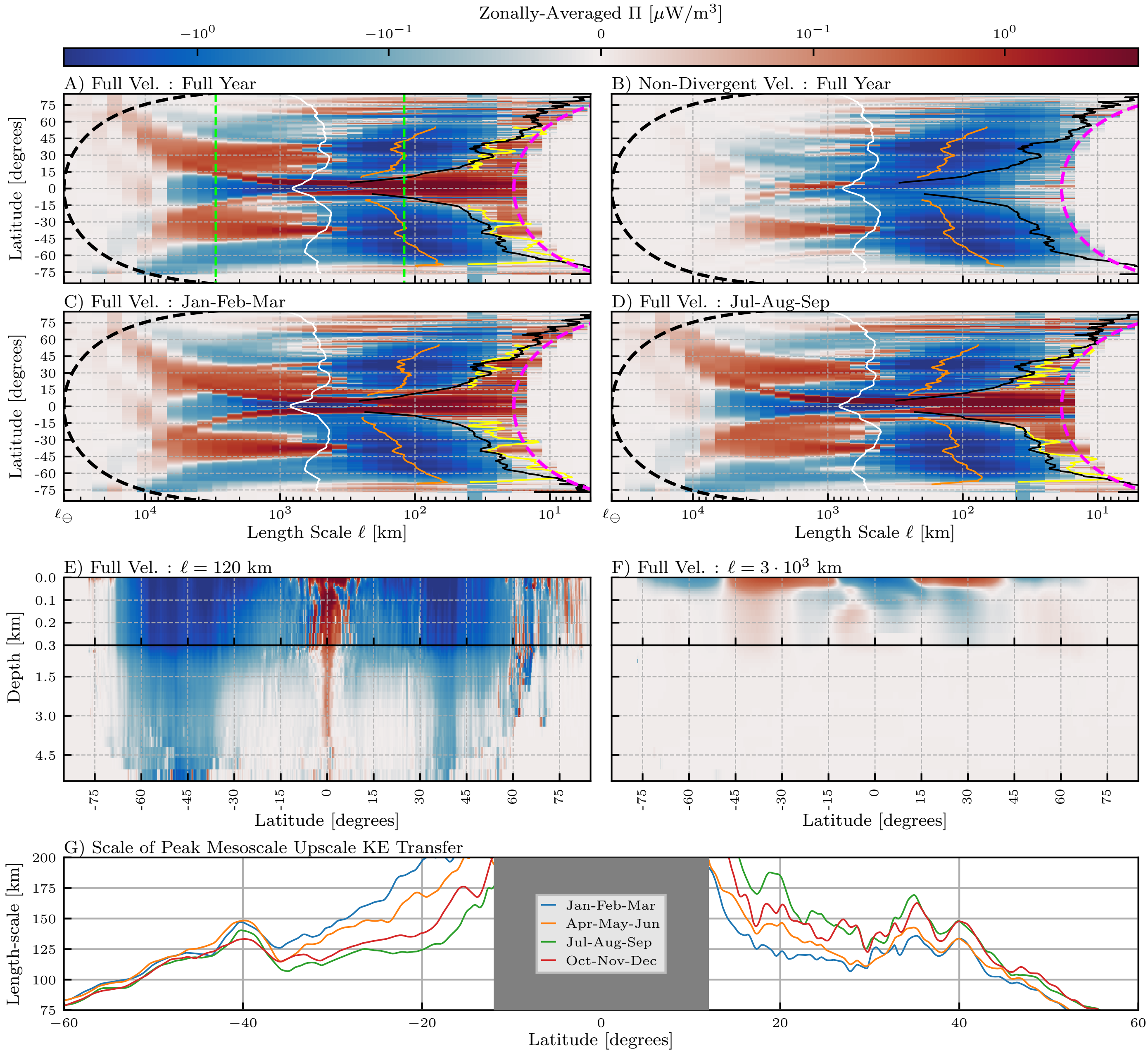

Figure S4: A reproduction of Figure 3, with panels [A-D] now included a solid white contour line that shows ℓ_{Rhines}.

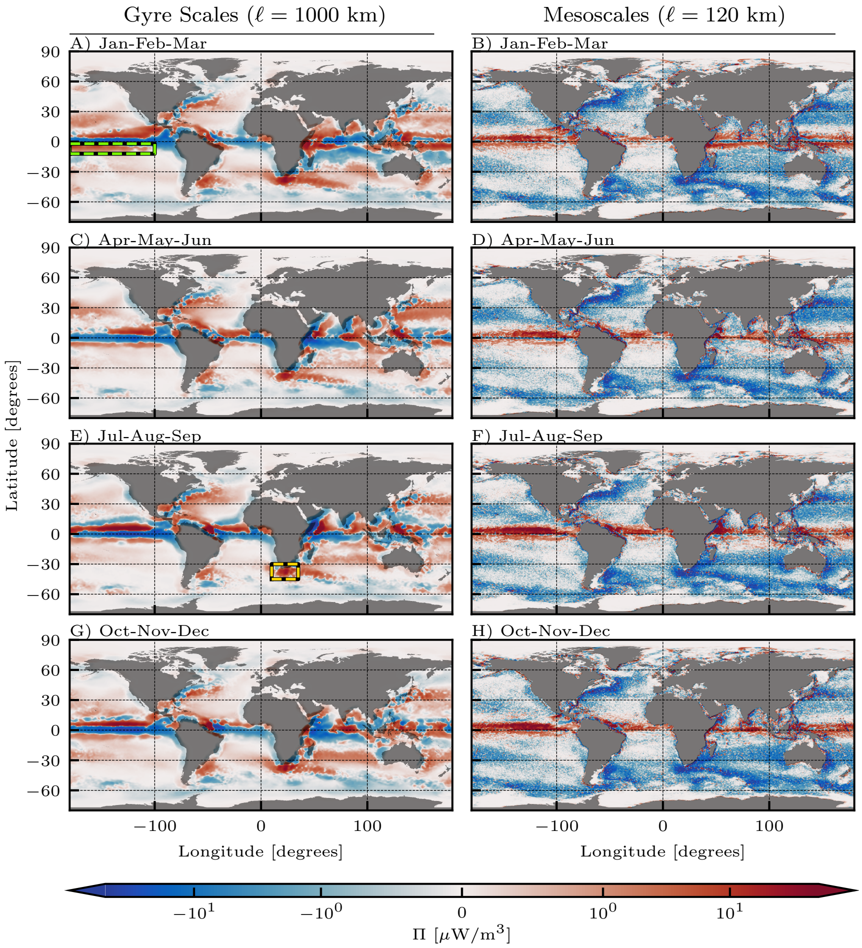

Figure S5: Π **Maps at all Seasons** Similar to Figure 2A-D, but showing all four seasons for both gyre-scale and mesoscale transfer: [A,B] Jan-Feb-Mar, [C,D] Apr-May-Jun, [E,F] Jul-Aug-Sep, and [G,H] Oct-Nov Dec for [A,C,E,G] $\ell = 1000$ km and [B,D,F,H] $\ell = 120$ km. All panels share a common colour bar, shown along the bottom of the figure. All panels show energy scale-transfer arising from the full velocity.

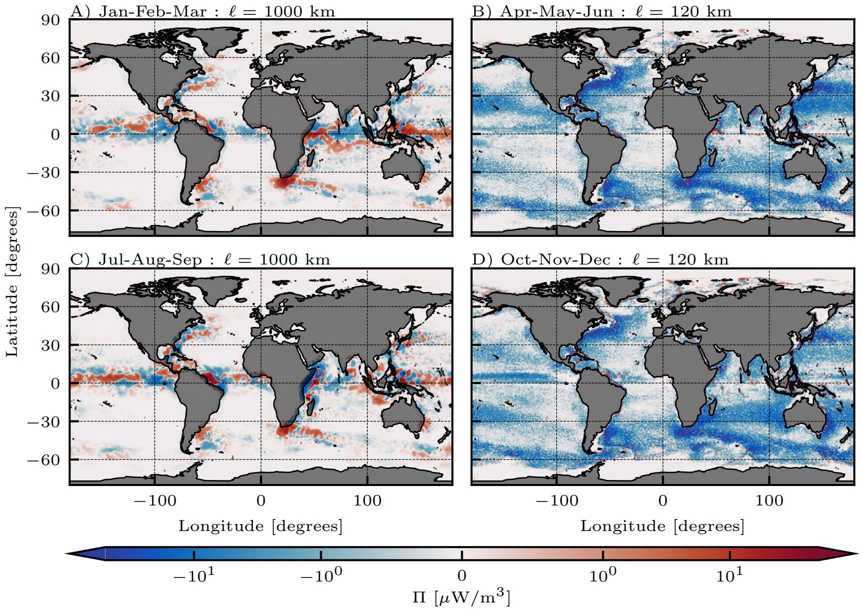

Figure S6: Π **Maps for Laterally Non-Divergent Flow** Similar to Figure 2A-D, but showing the energy scale-transfer arising solely from the laterally non-divergent flow component. Panels correspond to [A] Jan-Feb-Mar for $\ell = 1000$ km, [B] Apr-May-Jun for $\ell = 120$ km, [C] Jul-Aug-Sep for $\ell = 1000$ km, [D] Oct-Nov-Dec for $\ell = 120$ km. All panels share a common colour bar, shown along the bottom of the figure.

COMPARISON WITH AVISO KE SCALE-TANSFER

Figure S7 below is similar to Fig 3 in the main text and compares the scale-transfer from AVISO to that from NEMO using the laterally non-divergent velocity. Note that velocity field from the AVISO dataset is, by construction, approximately laterally non-divergent [5, 31] and does not incorporate the Ekman flow component of the oceanic circulation. Panels [A-B] in Figure S7 show similar Π patterns, with even the regions of downscale transfer agreeing well. Panel [C] also shows that in both AVISO and NEMO, the length-scale at which Π peaks generally decreases poleward except in strong current systems. There are two main differences between the NEMO and AVISO datasets: (i) AVISO has a lower Π magnitude and (ii) the peak Π occurs at larger scales (panels C-D). Both of these differences can be attributed to the effective smoothing and lower resolution of the AVISO dataset, as was discussed in prior work [28, 31]. These differences are also seen in the maps of Π using AVISO in Figure S8, where the gyre-scale KE transfer shows remarkable agreement with that from NEMO in Figure 2E, while the mesoscale cascade is weaker compared to that from NEMO in Figure 2F. Note that unlike KE scale-transfer, comparing KE *spectra* from NEMO and AVISO in [3] found a remarkably good agreement over all scales > 100 km.

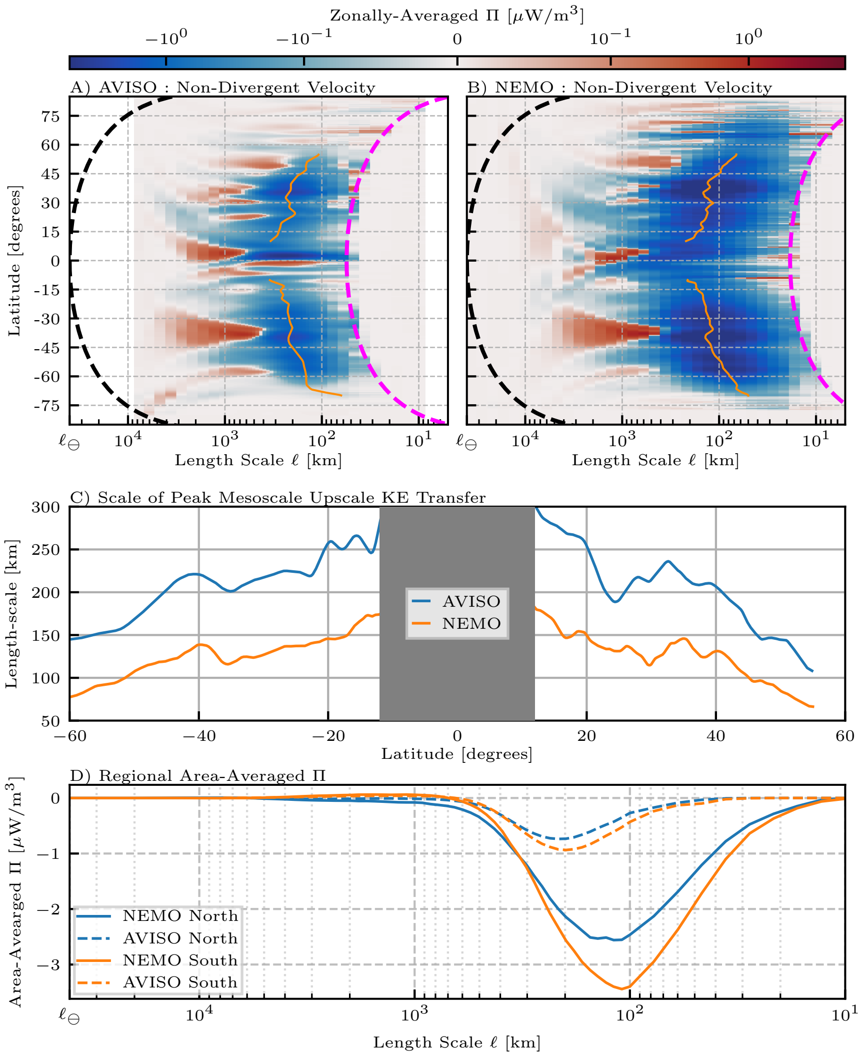

Figure S7: Comparison of KE Scale-Transfer between NEMO and AVISO Comparison of zonal means of Π between [A] AVISO and [B] NEMO, using the laterally non-divergent (toroidal) velocity components. In [A-B], orange lines show the scale with greatest magnitude, thick dashed black lines show the zonal circumference at each latitude, and thick dashed purple lines show the zonal length of two grid-points at each latitude. Panel [C] shows the length-scale of peak mesoscale inverse cascade from each of [A-B]. Panel [D] shows the area-averaged Π over the north ($[15°\mathrm{N}, 90°\mathrm{N}]$) and south ($[90°\mathrm{S}, 15°\mathrm{S}]$).

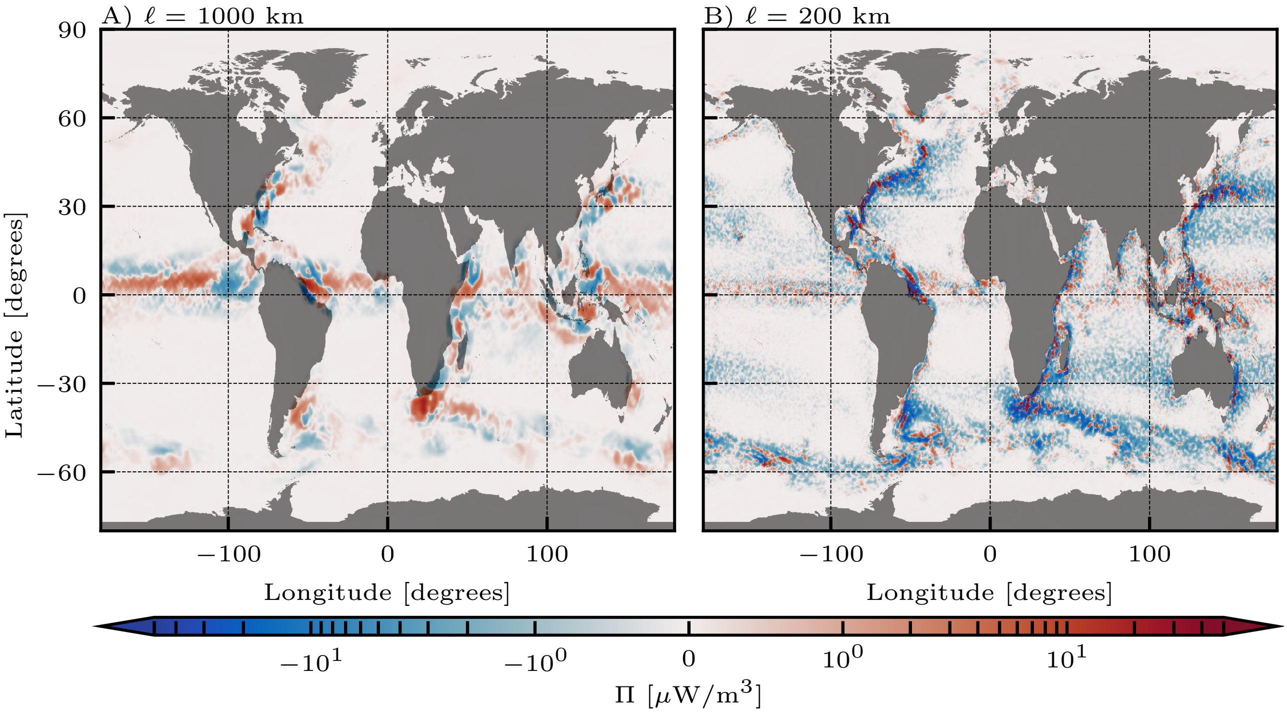

Figure S8: Maps of Π from AVISO Similar to Figure 2E-F, but showing the 9-year time-averaged Π obtained from the laterally non-divergent AVISO velocity for [A] 1000 km and [B] 200 km, which roughly corresponds to the peak cascade from Figure S7D. The colour bar is the same as the one used in Figure 2.
